# Caspases Switch off the m^6^A RNA Modification Pathway to Foster the Replication of a Ubiquitous Human Tumor Virus

**DOI:** 10.1128/mBio.01706-21

**Published:** 2021-08-24

**Authors:** Kun Zhang, Yucheng Zhang, Yunash Maharjan, Febri Gunawan Sugiokto, Jun Wan, Renfeng Li

**Affiliations:** a Philips Institute for Oral Health Research, School of Dentistry, Virginia Commonwealth University, Richmond, Virginia, USA; b Department of Oral and Craniofacial Molecular Biology, School of Dentistry, Virginia Commonwealth University, Richmond, Virginia, USA; c Department of Medical and Molecular Genetics, Indiana University School of Medicine, Indianapolis, Indiana, USA; d Center for Computational Biology and Bioinformatics, Indiana University School of Medicine, Indianapolis, Indiana, USA; e Department of BioHealth Informatics, School of Informatics and Computing, Indiana University–Purdue University at Indianapolis, Indianapolis, Indiana, USA; f Department of Microbiology and Immunology, School of Medicine, Virginia Commonwealth University, Richmond, Virginia, USA; g Massey Cancer Center, Virginia Commonwealth University, Richmond, Virginia, USA; University of North Carolina, Chapel Hill

**Keywords:** reactivation, lytic replication, restriction factor, m^6^A RNA modification, YTHDF2, METTL3, METTL14, WTAP, caspase cleavage, Epstein-Barr virus

## Abstract

The methylation of RNA at the N6 position of adenosine (m^6^A) orchestrates multiple biological processes to control development, differentiation, and cell cycle, as well as various aspects of the virus life cycle. How the m^6^A RNA modification pathway is regulated to finely tune these processes remains poorly understood. Here, we discovered the m^6^A reader YTHDF2 as a caspase substrate via proteome-wide prediction, followed by *in vitro* and *in vivo* validations. We further demonstrated that cleavage-resistant YTHDF2 blocks, while cleavage-mimicking YTHDF2 fragments promote, the replication of a common human oncogenic virus, Epstein-Barr virus (EBV). Intriguingly, our study revealed a feedback regulation between YTHDF2 and caspase-8 via m^6^A modification of *CASP8* mRNA and YTHDF2 cleavage during EBV replication. Further, we discovered that caspases cleave multiple components within the m^6^A RNA modification pathway to benefit EBV replication. Our study establishes that caspase disarming of the m^6^A RNA modification machinery fosters EBV replication.

## INTRODUCTION

Epstein-Barr virus (EBV) is a ubiquitous tumor virus causing several types of cancer of B cell and epithelial cell origins (1, 2). Globally, EBV infection causes more than 200,000 new cancer cases and 140,000 deaths per year (3). The life cycle of EBV include a quiescent latent phase and an active replication phase (4). The switch from latency to lytic replication, also called reactivation, involves a series of signaling pathways that drive the expression of two EBV immediate early genes, *ZTA* and *RTA* (5). Host factors that restrict or promote the expression of *ZTA* and *RTA* determine the threshold for EBV lytic cycle activation (5–8). Oncolytic therapies based on reactivation of latent virus are a promising approach for targeted treatment of EBV-associated cancers (9), but these strategies require a deeper understanding of how the EBV life cycle is dynamically regulated by key cellular processes and pathways.

The *N*^6^-methyladenosine (m^6^A) modification of viral and cellular mRNAs provides a novel mechanism of posttranscriptional control of gene expression (10–12). m^6^A modification is dynamically regulated by methyltransferases (writers; METTL3, METTL14, WTAP, and VIRMA) and demethylases (erasers; ALKBH5 and FTO) (10, 13–15). The m^6^A-specific binding proteins (readers; e.g., YTHDF1/2/3 and YTHDC1/2) regulate various aspects of RNA function, including stability, splicing, and translation (10, 11, 16, 17). YTHDF2 mainly regulates mRNA decay through direct recruitment of the CCR4-NOT deadenylase complex (16, 18). Recent studies showed that YTHDF1 and YTHDF3 share redundant roles with YTHDF2 in RNA decay (19, 20). All three YTHDF proteins undergo phase separation through binding to m^6^A-modified RNA (21–23).

The m^6^A RNA modification pathway has been shown to promote or restrict herpesvirus infection and replication depending on the viral and cellular context. The depletion of YTHDF1 and YTHDF2 has been shown to promote EBV lytic protein expression (24, 25). The EBV immediate early protein ZTA suppresses METTL3 expression through binding to its promoter (26), and the EBV latent protein EBNA3C activates METTL14 transcription and directly interacts with and stabilizes METTL14 to promote oncogenesis (25). Both EBV latent and lytic genes are modified by m^6^A during primary infection, latency, or lytic reactivation (25, 27). Recently, it was shown that YTHDF2 binding to m^6^A-modified viral RNA restricts Kaposi’s sarcoma-associated herpesvirus (KSHV) and EBV replication by promoting RNA decay (25, 28). However, another study showed that YTHDF2 either restricts or promotes KSHV replication depending on cell type (29). In addition, YTHDC1 was shown to bind to m^6^A-modified KSHV *RTA*/*ORF50* to regulate its pre-mRNA splicing and promote lytic replication (30). SND1 was recently discovered as another m^6^A reader that binds and stabilizes KSHV *RTA/ORF50* transcript to promote KSHV replication (31). m^6^A modification of cellular genes promotes human cytomegalovirus (HCMV) replication by downregulating the interferon pathway (32). The complex function of m^6^A pathway in herpesvirus infection suggests that this pathway must be finely regulated during viral infection or reactivation in different cellular contexts.

Although caspase-mediated cell death is intrinsically hostile to viral replication, emerging studies from our group and others have demonstrated that caspase cleavage of cellular restriction factors promotes EBV and KSHV reactivation (6, 7, 33–35). Based on two evolutionarily conserved cleavage motifs we discovered for PIAS1 protein regulation during EBV reactivation (7), we screened the entire human proteome and identified 16 potential caspase substates that carry the same motifs. Among these proteins, we validated 5 of 6 as bona fide caspase substrates and then focused on the m^6^A reader YTHDF2 and, subsequently, the entire m^6^A RNA modification pathway in the context of EBV reactivation process. We found that caspase-mediated cleavage converts YTHDF2 from a restriction factor to several fragments that promote EBV replication. Mechanistically, we demonstrated that YTHDF2 promotes the degradation of *CASP8* mRNA via m^6^A modification to limit caspase activation and viral replication. Importantly, we further illustrated that multiple m^6^A pathway components, including methyltransferases and other readers, were also cleaved by caspases to promote viral replication. Together, these findings uncovered an unexpected cross-regulation between caspases and the m^6^A RNA modification pathway in promoting EBV replication.

## RESULTS

### Cleavage sequence-guided screening identifies potential novel host factors involved in EBV replication.

**FIG 1:**
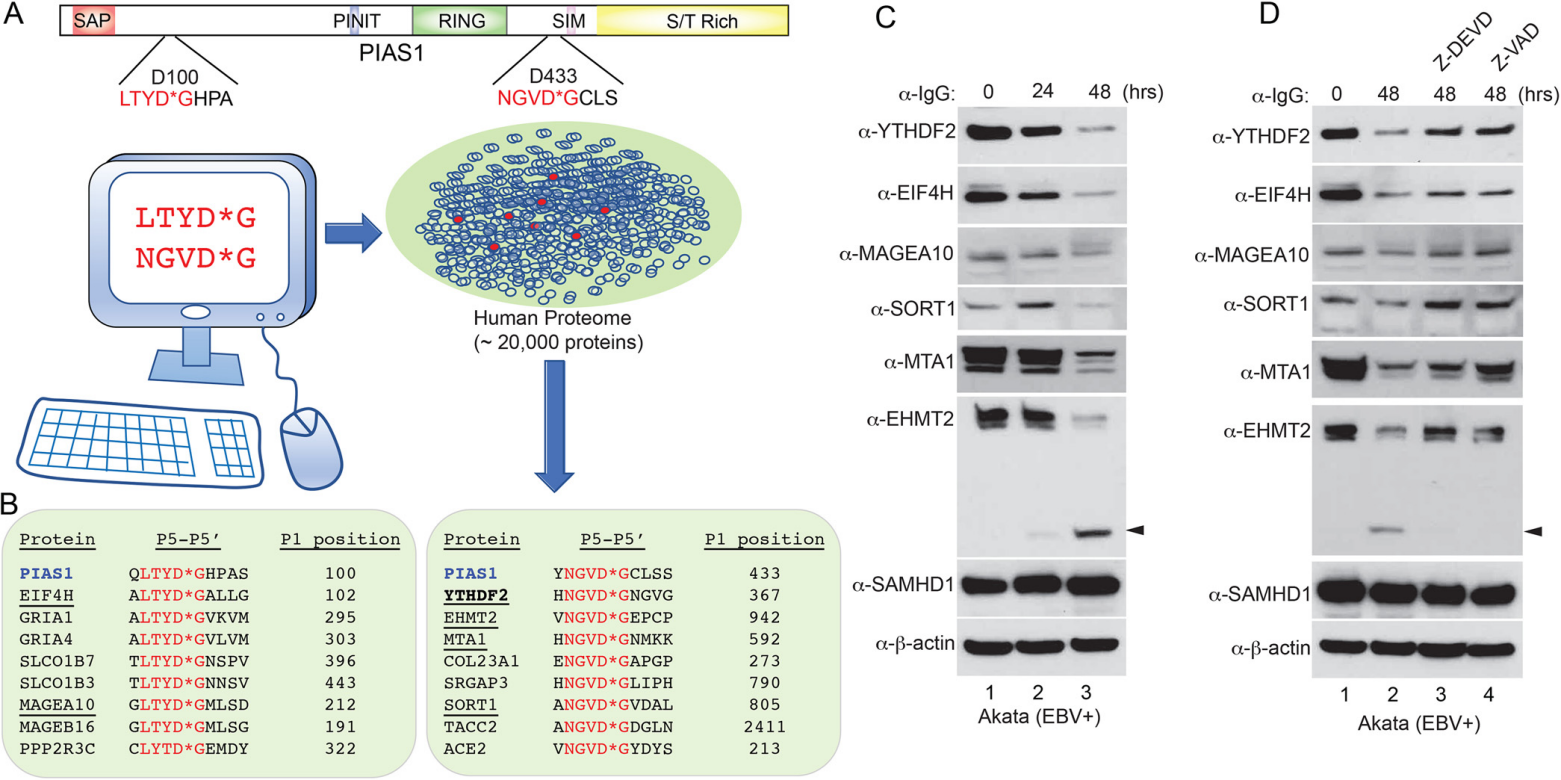
A virtual screen identified new potential caspase substrates during EBV reactivation. (A) The cleavage motifs derived from PIAS1 (LTYD*G and NGVD*G) were used to virtually screen the entire human proteome for proteins sharing the same sequences. The human proteome data set containing approximately 20,000 human protein-coding genes represented by the canonical protein sequence was downloaded from UniProtKB/Swiss-Prot. (B) Sixteen additional proteins were extracted from the screen. Eight carry the LTYD*G motif (left), and eight carry the NGVD*G motif (right). Six proteins (underlined) were selected for further validation. (C) Protein downregulation during EBV reactivation. Akata (EBV^+^) cells was treated with anti-IgG antibody to induce EBV reactivation for 0, 24, and 48 h. Western blot showing the downregulation of 6 selected proteins using antibodies as indicated. SAMHD1 and β-actin were included as controls. The arrowhead indicates the cleaved EHMT2 fragment. (D) Caspase inhibition blocks the degradation of YTHDF2, MAGEA10, SORT1, MTA1, and EHMT2. The Akata (EBV^+^) cells were either untreated or pretreated with a caspase-3/-7 inhibitor (Z-DEVD-FMK; 50 μM) or a pan-caspase inhibitor (Z-VAD-FMK; 50 μM) for 1 h, and then anti-IgG antibody was added for 48 h. Western blot showing the protein levels of 6 selected proteins using the indicated antibodies. SAMHD1 and β-actin were included as controls. The arrowhead indicates the cleaved EHMT2 fragment.

Recently, our group demonstrated that PIAS1 is an EBV restriction factor that is cleaved by caspase-3, -6, and -8 to promote EBV replication (7). The two evolutionally conserved sequences surrounding PIAS1 cleavage sites D100 and D433 (LTYD*G and NGVD*G) are quite different from the canonical caspase cleavage motifs (36). We predicted that other proteins within the cellular proteome may contain the same sequences and hence are potentially regulated by caspases. Using these two cleavage sequences to search the human proteome, we discovered 16 additional putative caspase substrates, including RNA binding proteins (YTHDF2 and EIF4H), chromatin remodeling proteins (MTA1 and EHMT2), and a severe acute respiratory syndrome coronavirus 2 (SARS-CoV2) receptor ACE2 (Fig. 1A and B).

We selected 6 candidates (YTHDF2, EIF4H, MAGEA10, SORT1, MTA1, and EHMT2) to monitor their protein levels in EBV-positive Akata cells upon anti-IgG-induced B cell receptor (BCR) activation, a process that triggers caspase activation and EBV reactivation (Fig. 1C) (6, 7, 37). All 6 proteins were downregulated upon BCR activation but not SAMHD1, a cellular restriction factor against EBV replication (38). To further determine whether any of these proteins are regulated by caspase, we pretreated the cells with a caspase-3/7 inhibitor or a pan-caspase inhibitor followed by IgG-cross-linking of BCR. We found that the downregulation of YTHDF2, MAGEA10, SORT1, MTA1, and EHMT2 was reversed by the caspase inhibition, suggesting that these proteins are bona fide caspase substrates in cells (Fig. 1D). Caspase inhibition partially restored EIF4H protein expression, indicating an involvement of other pathways in controlling EIF4H protein level (Fig. 1D).

**FIG 2:**
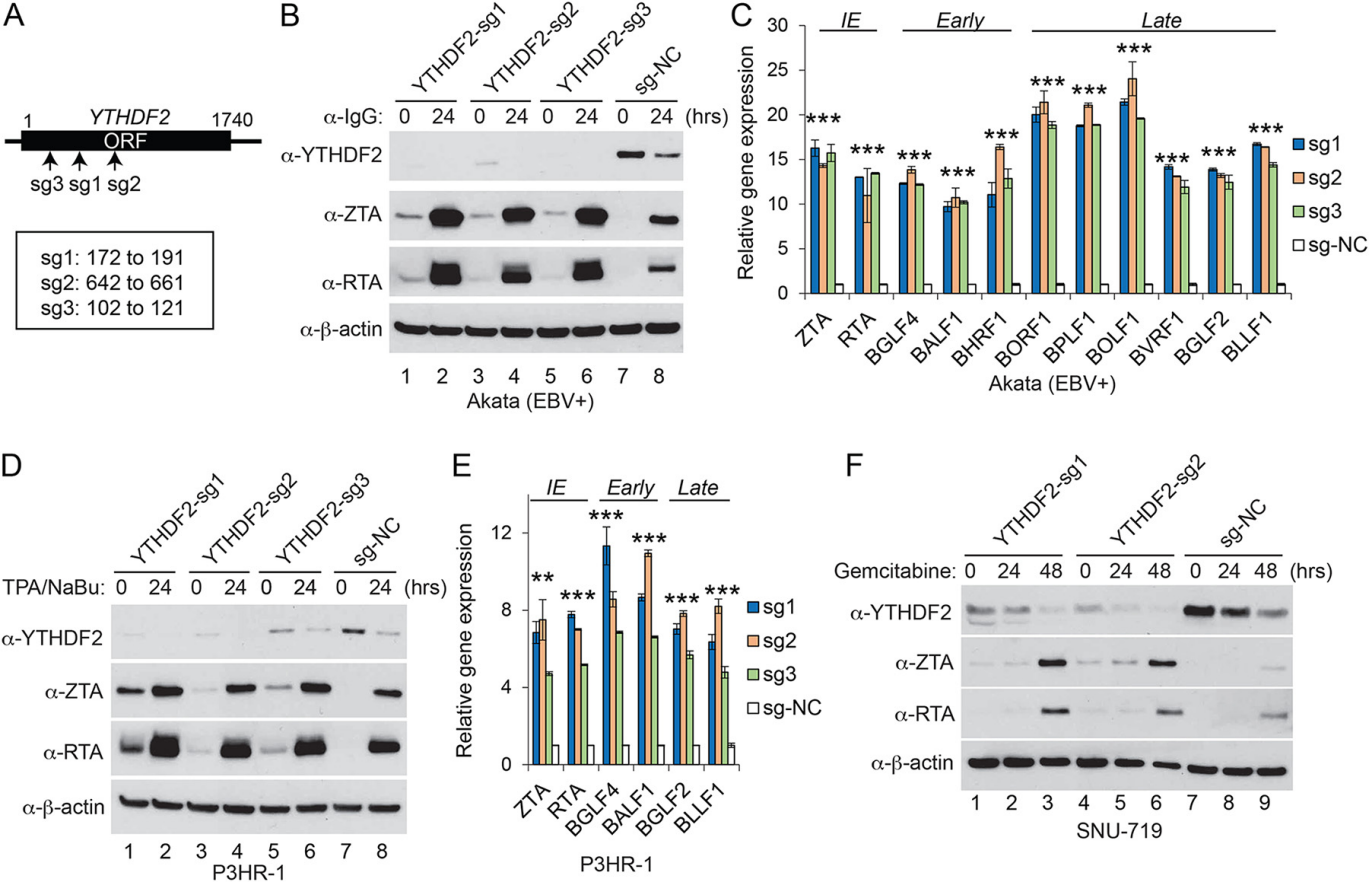
YTHDF2 restricts EBV reactivation. (A) Schematic representation showing the relative positions of Cas9 target sites for small guide RNAs sg1 to sg3. (B) Akata (EBV^+^) cells were used to establish stable cell lines using 3 different sgRNA constructs and a nontargeting control (sg-NC). The cells were untreated or lytically induced with anti-IgG-mediated cross-linking of BCR. YTHDF2 and viral protein (ZTA and RTA) expression levels were monitored by Western blotting using the indicated antibodies. (C) RNAs from YTHDF2-depleted and control Akata cells were extracted and analyzed by RT-qPCR. The values for the control were set as 1. Error bars indicate SD. IE, immediate early gene; Early, early gene; Late, late gene. (D) P3HR-1 cells were used to establish stable cell lines as indicated. The cells were either untreated or treated with TPA and sodium butyrate (NaBu) to induce lytic reactivation. YTHDF2 and viral protein expression levels were monitored by Western blotting using the indicated antibodies. (E) RNAs from YTHDF2-depleted and control P3HR-1 cells were extracted and analyzed by RT-qPCR. The values for the control were set as 1. Error bars indicate SD. IE, immediate early gene; Early, early gene; Late, late gene. (F) SUN-719 cells were used to establish stable cell lines as indicated. The cells were either untreated or treated with gemcitabine to induce lytic reactivation. YTHDF2 and viral protein expression levels were monitored by Western blotting using the indicated antibodies. Results from three biological replicates are presented. Error bars indicate SD. **, *P*< 0.01; ***, *P*< 0.001.

To determine whether any of these proteins play a role in EBV life cycle, we utilized a CRISPR/Cas9 genome editing approach to knock out the corresponding genes in Akata (EBV^+^) cells, and then evaluated the viral lytic gene products upon BCR stimulation. The depletion of YTHDF2 and EIF4H promoted EBV ZTA and RTA protein expression (Fig. 2A and B; also, see Fig. S1A in the supplemental material). In contrast, the depletion of MAGEA10, SORT1, EHMT2, and MTA1 did not affect EBV protein expression (Fig. S1B to E).

10.1128/mBio.01706-21.1 FIG S1 The role of EIF4H, MAGEA10, SORT1, EHMT2, MTA1 and YTHDF2 during EBV lytic induction. See also Fig. 2. (A to E) Roles of EIF4H, MAGEA10, SORT1, EHMT2, and MTA1 during EBV lytic induction. Akata (EBV^+^) cells were used to establish stable cell lines using 2 or 3 different sgRNA constructs and a nontargeting control (sg-NC). The cells were untreated or lytically induced with anti-IgG treatment for 24 or 48 h, as indicated. Cellular and viral protein expression levels were monitored by Western blotting using the indicated antibodies. (A) EIF4H depletion promotes the expression of EBV ZTA and RTA. (B) MAGEA10 depletion does not affect EBV protein expression. (C) SORT1 depletion does not significantly affect EBV protein expression. (D) EHMT2 depletion does not affect EBV protein expression. The arrowhead indicates cleaved fragments. (E) MTA1 depletion does not uniformly affect EBV protein expression but slightly enhances the expression of its homolog MTA2. (F to H) YTHDF2 depletion promotes EBV lytic replication. (F) YTHDF2-depleted and control Akata (EBV^+^) cells were lytically induced with anti-IgG for 0 to 48 h. (G) YTHDF2-depleted and control P3HR1 cells were lytically induced with TPA and NaBu for 0 to 48 h. (H) YTHDF2-depleted and control SNU-719 cells were lytically induced with TPA and NaBu for 0 to 48 h. Extracellular virion DNA from the medium was extracted and then analyzed by qPCR using primers specific to BALF5. The value of the vector control at 0 h was set as 1. Results from three biological replicates are presented. Error bars indicate SD. ND, not detected. *, *P*< 0.05; **, *P*< 0.01; ***, *P*< 0.001. Download FIG S1, TIF file, 1.5 MB. Copyright © 2021 Zhang et al. 2021 Zhang et al. https://creativecommons.org/licenses/by/4.0/ This content is distributed under the terms of the Creative Commons Attribution 4.0 International license.

In addition, we demonstrated that YTHDF2 depletion promoted the expression of immediate early, early, and late genes even without lytic induction (Fig. 2C), suggesting that YTHDF2 serves as a restriction factor against EBV reactivation under normal conditions. Due to the key role of the m^6^A RNA modification pathway in viral life cycle, we selected the m^6^A reader YTHDF2 as a starting point to explore the regulation of this pathway by caspases in EBV replication.

### YTHDF2 universally restricts EBV replication.

YTHDF2 has been shown to promote or suppress KSHV lytic replication in a cell type-dependent manner but mainly to inhibit EBV replication in Burkitt lymphoma cells (25, 28, 29) (Fig. 2B and C). To investigate whether YTHDF2 has a unified function in EBV reactivation regardless of cell type and lytic induction methods, we knocked out YTHDF2 in another Burkitt lymphoma cell line, P3HR-1, and a gastric cancer cell line, SNU-719. We found that YTHDF2 depletion promoted EBV gene expression in both cell lines using two different lytic inducers (Fig. 2D to F), demonstrating a universal role of YTHDF2 in suppressing EBV gene expression. To determine whether YTHDF2 plays a role in EBV life cycle, we monitored the virion released to the medium upon lytic induction. We observed that YTHDF2 depletion significantly enhanced EBV copy numbers in Akata (EBV^+^), P3HR-1, and SNU-719 cells upon lytic induction (Fig. S1F to H), suggesting that YTHDF2 restricts EBV replication regardless of cell type.

### YTHDF2 is cleaved by caspase-3, -6, and -8 at two evolutionarily conserved sites.

**FIG 3:**
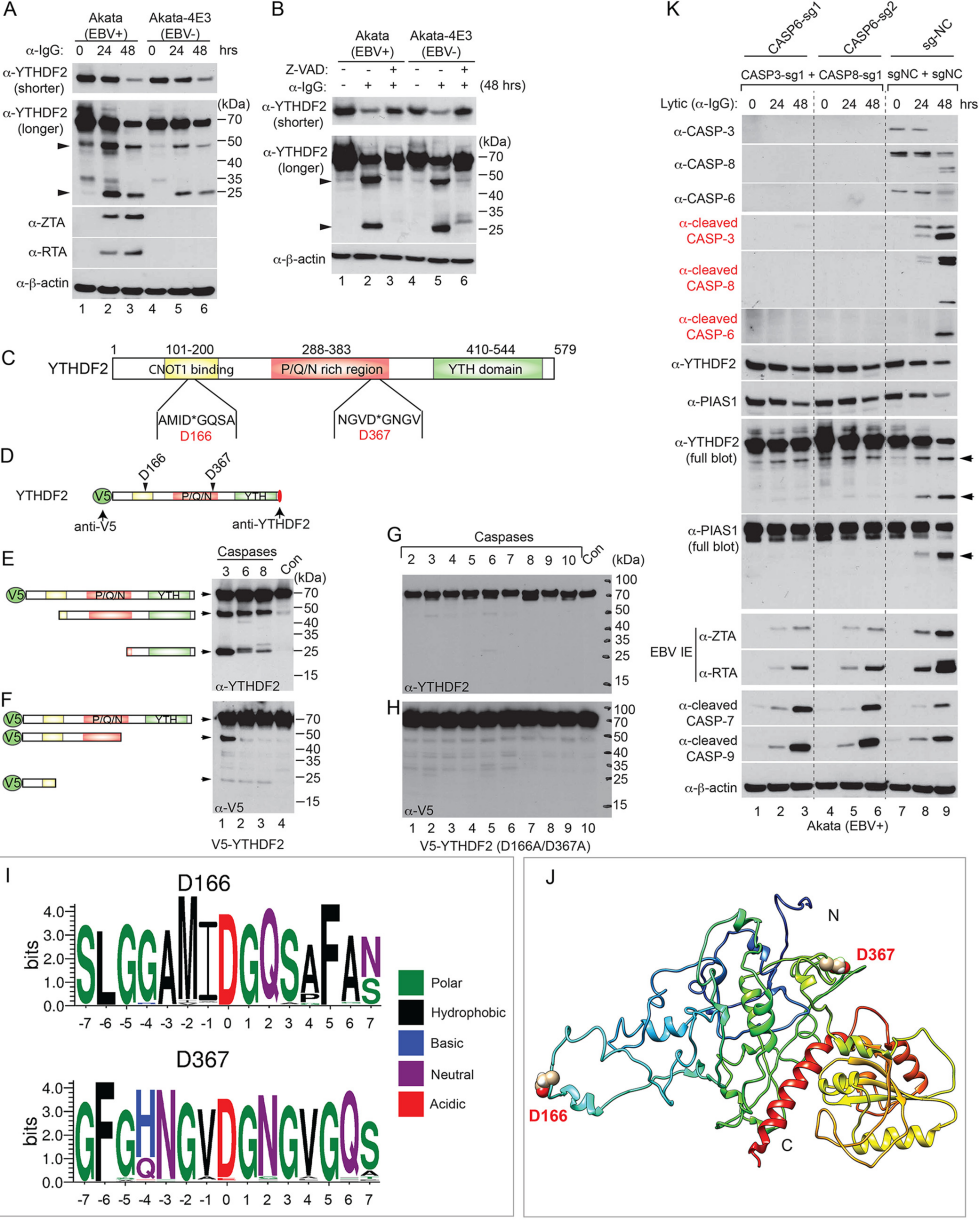
YTHDF2 is cleaved by caspases *in vivo* and *in vitro*. (A) Western blot showing YTHDF2 downregulation by IgG cross-linking-induced BCR activation. Akata (EBV^+^) and Akata-4E3 (EBV^−^) cells were treated with anti-IgG antibody as indicated. YTHDF2 and viral protein expression levels were monitored by Western blotting. Arrowheads indicate cleaved YTHDF2 in the longer-exposure blot. (B) Caspase inhibition blocks YTHDF2 degradation. The cells were either untreated or pretreated with a pan-caspase inhibitor (Z-VAD-FMK; 50 μM) for 1 h, and then anti-IgG antibody was added for 48 h. Arrowheads indicate cleaved YTHDF2. (C) Functional domains and putative cleavage sites in YTHDF2. CaspDB was used to predict the potential cleavage sites in YTHDF2. The locations of the putative cleavage sites D166 and D367 are indicated. The CNOT1 binding domain is responsible for the degradation of associated RNA; the P/Q/N-rich region is an aggregation-prone region; the YTH domain is responsible for binding to m^6^A-modified RNA. (D) Schematic representation of V5-tagged YTHDF2 with two putative cleavage sites. Red oval, anti-YTHDF2 monoclonal antibody recognition site. (E and F). Wild-type V5-YTHDF2 was incubated with individual recombinant caspase for 2 h. Western blotting was performed using either anti-YTHDF2 (E) or anti-V5 (F) antibodies. The relative positions of predicted cleavage fragments are indicated. (G and H) YTHDF2 (D166A/D367A) mutant protein was incubated with individual recombinant caspase for 2 h. Western blotting was performed using the indicated antibodies. (I) Motif analysis showing the conservation of the two cleavage sites and the surrounding amino acids. Amino acid sequences were extracted from 97 (D166) and 80 (D367) vertebrate species, and motif logos were generated using WebLogo. (J) Structure modeling of full-length YTHDF2 by I-TASSER. The cleavage sites D166 and D367 are labeled. N and C denote N terminus and C terminus, respectively. (K) Triple depletion of caspase-3, -8, and -6 reduces YTHDF2 and PIAS1 degradation and blocks viral protein accumulation. The CASP3/CASP8/CASP6-depleted Akata (EBV^+^) cells were lytically induced by anti-IgG treatment. The expression of caspases, cleaved caspases, YTHDF2, PIAS1, and viral proteins (ZTA and RTA) was monitored by Western blotting using the indicated antibodies. Arrowheads indicate cleaved fragments.

Having shown YTHDF2 as a caspase substrate during the course of EBV replication (Fig. 1B and C), we further observed a downregulation of YTHDF2 in Akata-4E3 (EBV^−^) cells upon IgG cross-linking, suggesting that this physiologically relevant lytic trigger plays a major role in YTHDF2 destabilization (Fig. 3A, YTHDF2 shorter exposure, lanes 4 to 6). In addition to the reduction of protein level, two putative cleaved fragments of YTHDF2 were observed, whose molecular weights (MWs) are 25 and 50 kDa, respectively (Fig. 3A, YTHDF2 longer exposure).

As expected, caspase inhibition also blocked the generation of two fragments in both Akata (EBV^+^) and Akata-4E3 (EBV^−^) cells (Fig. 3B, YTHDF2 longer exposure). We predicted that the 25-kDa fragment likely represents a C-terminally cleaved YTHDF2 (amino acids [aa] 368 to 579; predicted MW, 24 kDa). Similarly, YTHDF2 was also downregulated in lytically induced P3HR-1 (Fig. S2A, lanes 1 and 2) and SNU-719 cells (Fig. S2B, lanes 1 and 2). Caspase inhibition blocked YTHDF2 degradation and inhibited viral protein expression (Fig. S2A and B, lanes 3 and 4) (7).

10.1128/mBio.01706-21.2 FIG S2 YTHDF2 is cleaved by caspases to promote EBV replication. See also Fig. 3 and Table S1B. (A) Caspase inhibition blocks YTHDF2 degradation and viral protein expression. P3HR1 cells were either untreated or pretreated with caspase-3 (Z-DEVD-FMK) or pan-caspase inhibitors (Z-VAD-FMK) (50 μM) for 1 h and then lytically induced by TPA and sodium butyrate (NaBu) for 48 h. YTHDF2 and EBV ZTA/RTA protein levels were monitored by Western blotting. (B) Caspase inhibition blocks YTHDF2 degradation in SNU-719 cells. SNU-719 cells were either untreated or pretreated with caspase-3 or pan-caspase inhibitors (50 μM) for 1 h and then lytically induced by gemcitabine (1 μg/ml) for 48 h. YTHDF2 and EBV ZTA/RTA protein levels were monitored with the indicated antibodies. (C) Recombinant wild-type YTHDF2 was incubated with individual caspases for 2 h at 37°C. Western blot analysis showing YTHDF2 cleavage by caspase-3, -6, -8, and to a lesser extent, caspase-7. Arrowheads denote cleavage fragments. (D) Sequence alignment of YTHDF2 sequences from 10 representative species using the constraint-based multiple alignment tool (COBALT). The cleavage motifs are highlighted in yellow. (E to G) Caspase-3/-6/-8 triple knockout suppresses EBV lytic replication. The CASP3/CASP8/CASP6-depleted Akata (EBV^+^) cells were lytically induced by anti-IgG treatment. (E and F) Total RNA was extracted, and then EBV ZTA and RTA mRNA levels were analyzed by RT-qPCR. (G) Extracellular virion DNA from the medium was extracted and then analyzed by qPCR using primers specific to BALF5. The value for the control at 0 h was set as 1. Results from three biological replicates are presented. Error bars indicate SD. ***, *P*< 0.001. Download FIG S2, TIF file, 1.0 MB. Copyright © 2021 Zhang et al. 2021 Zhang et al. https://creativecommons.org/licenses/by/4.0/ This content is distributed under the terms of the Creative Commons Attribution 4.0 International license.

To determine the major caspases responsible for YTHDF2 cleavage, we performed an *in vitro* cleavage assay using individual recombinant caspases and YTHDF2. We found that caspase-3, -6, and -8 and, to a lesser extent, caspase-7 all cleaved YTHDF2 (Fig. S2C). The sizes of cleaved fragments *in vitro* were similar to those observed in cells, suggesting that YTHDF2 is a bona fide caspase substrate *in vitro* and *in vivo*.

In addition to the predicted cleavage site D367 (Fig. 1A), we used an *in silico* prediction algorithm (CaspDB) to predict additional cleavage sites (39). A second-highest-scoring cleavage site (D166) was identified near the N terminus of YTHDF2 (Fig. 3C).

To explore whether there are two cleavage sites in YTHDF2, we first generated an N-terminally V5-tagged YTHDF2 by a method we used previously for PIAS1 (7), which allows the determination of both N- and C-terminal cleavage fragments by anti-V5 and anti-YTHDF2 (C-terminal) monoclonal antibodies, respectively (Fig. 3D). Given the sizes of the cleaved bands recognized by the C-terminal YTHDF2 antibody, we noticed that caspase-3-, -6-, and -8-mediated cleavage led to one ∼50-kDa C-terminal fragment and that caspase-3 and to a lesser extent caspase-6 and -8 generated one 25-kDa C-terminal fragment (Fig. 3E). The anti-V5 antibody revealed one ∼50-kDa N-terminal cleavage fragment generated by caspase-3 and one smaller N-terminal (slightly less than 25 kDa) fragment generated by all 3 caspases (Fig. 3F). Together, these results demonstrate that YTHDF2 is indeed cleaved at two sites by caspase-3, -6, and -8.

To further confirm whether D166 and D367 are the major cleavage sites, we mutated these two residues to alanines (D166A and D367A) and then examined the cleavage profile using *in vitro* cleavage assays (Fig. 3G and H). Consistent with our prediction, mutations at those two sites prevented YTHDF2 cleavage by caspases, indicating that D166 and D367 are the major cleavage sites. To examine the conservation across different species of these two cleavage motifs (AMID*G and NGVD*G) within YTHDF2, we extracted 15 amino acids surrounding the cleavage sites from 80 to 97 vertebrate species. Interestingly, we found that not only the cleavage motifs but also the surrounding amino acids are highly conserved (Fig. 3I; Fig. S2D), suggesting a key regulatory function for YTHDF2 cleavage during evolution.

Considering that cleavage sites are normally exposed to the surface of the protein, we used an I-TASSER (iterative threading assembly refinement) algorithm (40, 41) to generate a 3-dimensional (3D) structure for full-length YTHDF2 by integrating the crystal structure of the YTH domain (42, 43). One structure with two cleavage sites located on the protein surface was visualized with Chimera (44), and both sites fall within flexible regions favoring cleavage by caspases (Fig. 3J).

Because caspase-3, -6, and -8 are the major caspases that can redundantly cleave YTHDF2 (Fig. S2C) and another restriction factor, PIAS1 (7), we created two *CASP3/CASP8/CASP6* (genes encoding caspase-3, -8 and -6) triply depleted Akata (EBV^+^) cell lines by CRISPR/Cas9 approaches (Fig. 3K; total and cleaved caspase-3, -8, and -6 blots). The depletion of these three caspases alleviated the degradation of YTHDF2 and PIAS1 upon lytic induction (Fig. 3K, YTHDF2 and PIAS1 blots, lanes 2 and 3 and lanes 5 and 6 versus lanes 8 and 9). Consequently, the gene expression and protein accumulation of EBV ZTA and RTA were also significantly reduced in *CASP3/CASP8/CASP6-*depleted cells (Fig. S2E and F; Fig. 3K, ZTA and RTA blots, lanes 2 and 3 and lanes 5 and 6 versus lanes 8 and 9). As expected, caspase depletion also suppressed the production of EBV progeny released from the cells (Fig. S2G).

Interestingly, the depletion of caspase-3, -6, and -8 led to a slight increase of caspase-7 and -9 activation, which may contribute to the partial destabilization of YTHDF2 and PIAS1 upon lytic induction (Fig. 3K, YTHDF2 and PIAS1 blots, lanes 1 versus 3 and 4 versus 6).

These results suggested that lytic induction induced caspase activation and the subsequent cleavage of host restriction factors YTHDF2 and PIAS1 promotes EBV lytic replication (7).

### Caspase-mediated YTHDF2 cleavage promotes EBV replication.

**FIG 4:**
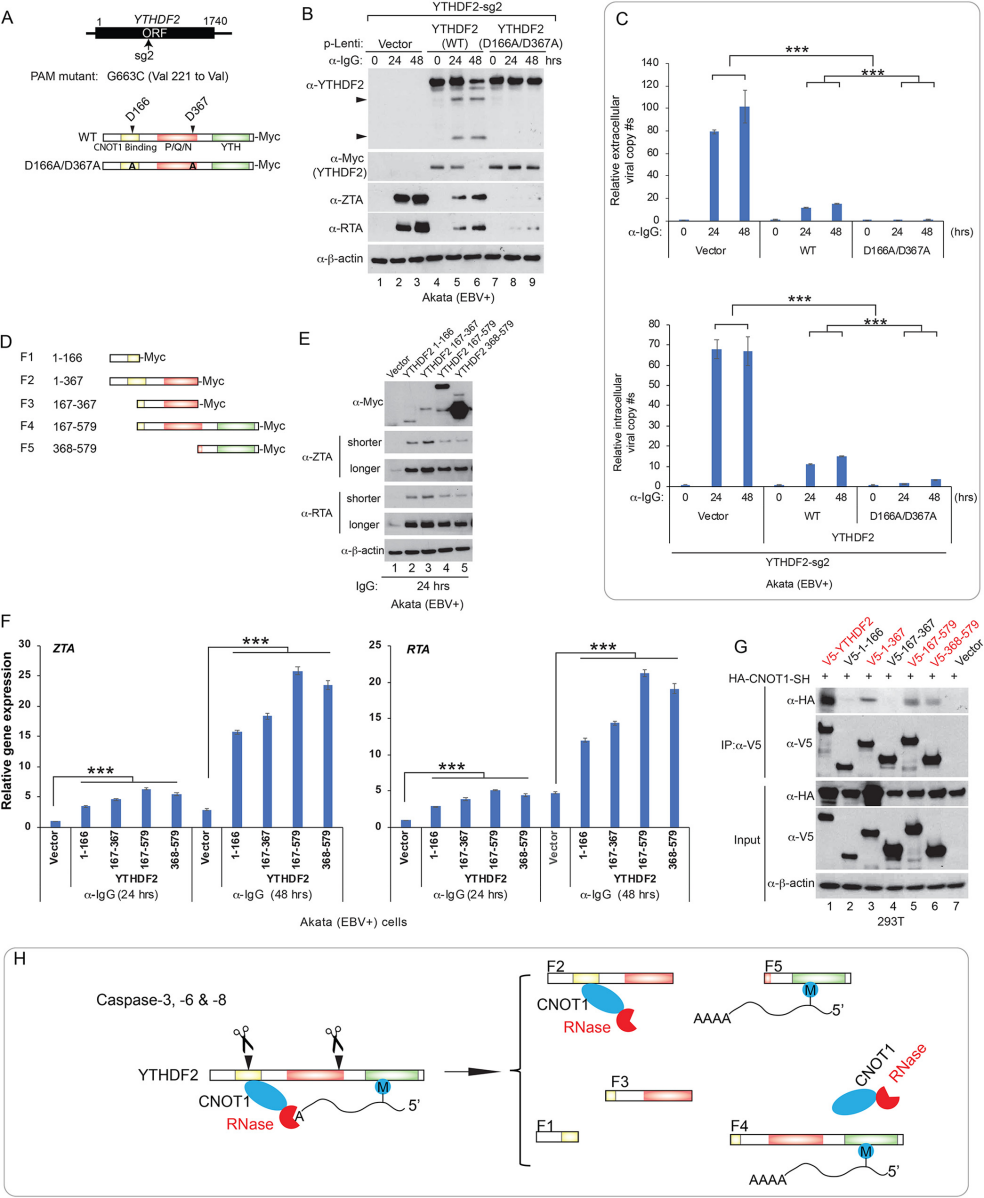
YTHDF2 cleavage promotes EBV replication. (A) The design of CRISPR/Cas9-resistant YTHDF2 variant was based on the sg2 protospacer-adjacent motif (PAM). D166A/D367A mutations were introduced into the PAM-mutated YTHDF2. Both constructs were cloned into a lentiviral vector with a C-terminal Myc tag. (B and C) WT and cleavage-resistant YTHDF2 suppresses EBV replication. Akata (EBV+) YTHDF2-sg2 cells carrying vector control, WT or cleavage-resistant YTHDF2 (D166A/D367A) were created using lentiviral transduction. Western blot analysis showing YTHDF2 and EBV protein expression levels in these cell lines upon IgG cross-linking (B). Arrowheads indicate cleaved fragments. Extracellular and intracellular viral DNA was measured by qPCR using primers specific to BALF5 (C). The value of the vector control at 0 h was set as 1. Results from three biological replicates are presented. Error bars indicate SD. **, *P* < 0.01; ***, *P* < 0.001. (D) Schematic representation of 5 YTHDF2 cleavage-mimicking fragments. These fragments were cloned into a lentiviral vector with a C-terminal Myc tag. (E and F) Akata (EBV^+^) cells were transduced with lentiviruses carrying vector control or individual fragments to establish stable cell lines. Western blot analysis showing YTHDF2 fragments and EBV protein expression levels in these cell lines upon lytic induction by anti-IgG treatment for 24 h (E). Shorter and longer exposures were included to show the differences in protein levels. RT-qPCR analysis showing EBV ZTA and RTA mRNA levels in these cell lines upon lytic induction by anti-IgG treatment for 24 and 48 h (F). The value of the vector control at 24 h was set as 1. Results from three biological replicates are presented. Error bars indicate SD. ***, *P* < 0.001. (G) Caspase-mediated cleavage impairs YTHDF2 binding to CNOT1. Halo-V5-tagged WT YTHDF2 and the individual fragments were cotransfected with the HA-tagged CNOT1 SH domain into 293T cells. Coimmunoprecipitation (IP) experiments were performed using anti-V5 antibody-conjugated magnetic beads. The immunoprecipitated samples and total cell lysates (Input) were analyzed by Western blotting with the indicated antibodies. (H) Model showing the functional consequences of YTHDF2 cleavage in CNOT1 binding and the targeting of m^6^A-modified RNA.

To further investigate whether caspase-mediated degradation of full-length YTHDF2 abrogates its restriction of EBV lytic replication, we first generated a CRISPR-resistant YTHDF2 variant in a lentiviral vector. This was achieved by introducing a silent mutation in the YTHDF2-sg2 PAM sequence (Fig. 4A) (7, 38). We first transduced the Akata (EBV^+^) cells with lentiviruses carrying vector control and wild-type (WT) and mutant (D166A/D367A) YTHDF2 to establish cell lines. We then depleted endogenous YTHDF2 by lentiviral transduction of YTHDF2-sg2 into these three cell lines. We then measured YTHDF2 protein degradation, viral protein accumulation, and viral DNA replication after lytic induction. The YTHDF2 (D166A/D367A) mutant was resistant to caspase-mediated cleavage and therefore became more stable than the WT counterpart upon lytic induction (Fig. 4B, lane 6 versus 9). Compared to WT YTHDF2, the D166A/D367A mutant strongly suppressed EBV protein accumulation (Fig. 4B) and consequently reduced the extracellular and intracellular viral copy numbers upon lytic induction (Fig. 4C), further consolidating caspase-mediated cleavage in antagonizing the antiviral function of YTHDF2.

Caspase-mediated cleavage led to not only a decrease of total YTHDF2 protein level but also an increase of cleaved fragments (Fig. 3A and B). We hypothesized that these cleavage fragments compete with WT YTHDF2 to promote viral replication. To test this hypothesis, we created a series of cleavage-mimicking fragments using a lentiviral vector (Fig. 4D). We then established fragment-expressing cell lines using Akata (EBV^+^) cells. An F2 (aa 1 to 367)-expressing cell line failed to become established after multiple attempts. However, in four other cell lines established, these cleavage-mimicking fragments strongly promoted EBV ZTA/RTA protein and *ZTA/RTA* mRNA expression (Fig. 4E, lane 1 versus 2 to 5, and Fig. 4F).

To further determine whether these results are due to protein overexpression, we created a pLenti-Halo control and transduced Akata (EBV^+^) cells to establish a Halo-expressing cell line. We also included vector control, WT YTHDF2-expressing, and F1 (aa 1 to 166)/F4 (aa 167 to 579)-expressing cell lines for comparison. We found that Halo expression had effects similar to those of the vector control in EBV ZTA and RTA protein expression (Fig. S3A, lane 1 versus 2 and lane 6 versus 7) and that WT YTHDF2 suppressed but F1 (aa 1 to 166) and F4 (aa 167 to 579) promoted the expression of EBV ZTA and RTA upon lytic induction (Fig. S3A, lanes 1 and 2 versus 3 to 5 and lanes 6 and 7 versus 8 to 10). As expected, we observed that viral RNA level correlated well with viral protein expression in these cell lines (Fig. S3B).

10.1128/mBio.01706-21.3 FIG S3 YTHDF2 cleavage fragments promotes EBV lytic gene expression. See also Fig. 4. (A) Akata (EBV^+^) cells were transduced with lentiviruses carrying vector control, Halo-tag, WT YTHDF2 or individual YTHDF2 fragment to establish stable cell lines. Western blot analysis showing Halo, YTHDF2 fragments and EBV protein expression levels in these cell lines upon lytic induction by anti-IgG treatment for 24 and 48 h. Shorter and longer exposures were included to show the differences in protein levels. (B) Total mRNA was extracted from cells treated in panel A. EBV ZTA and RTA mRNA levels were analyzed by RT-qPCR. The value of the vector control at 24 h was set as 1. Results from three biological replicates are presented. Error bars indicate SD. N.S., not significant; ***, *P*< 0.001. Download FIG S3, TIF file, 0.6 MB. Copyright © 2021 Zhang et al. 2021 Zhang et al. https://creativecommons.org/licenses/by/4.0/ This content is distributed under the terms of the Creative Commons Attribution 4.0 International license.

YTHDF2 has been shown to bind CNOT1 to recruit CCR4-NOT deadenylase complex for RNA decay (18). To determine whether YTHDF2 cleavage impairs its binding to CNOT1, we cotransfected a CNOT1-SH domain-expressing construct with an individual YTHDF2 fragment-expressing vector into 293T cells and then performed a coimmunoprecipitation (co-IP) assay. As expected, we found that CNOT1 is coimmunoprecipitated with WT YTHDF2 (Fig. 4G, lane 1). We also observed weaker binding signals of CNOT1 with F2 (aa 1 to 367), F4 (aa 167 to 579), and F5 (aa 368 to 579) fragments than with full-length YTHDF2 (Fig. 4G, lane 1 versus 3, 5, and 6). This is in part consistent with a previous study showing that YTHDF2 (aa 100 to 200) binds to CNOT1 (18). The binding between CNOT1 and F4 (aa 167 to 579)/F5 (aa 368 to 579) fragments could be mediated by a bridge protein or RNA. However, our study indicated that the YTH domain coordinates with the N-terminal region for enhanced YTHDF2 binding to CNOT1. Interestingly, two fragments, F1 (aa 1 to 166) and F3 (aa 167 to 367), lost the interaction with CNOT1, suggesting an essential N-terminal binding site for CNOT1 located near the caspase cleavage site D166 of YTHDF2 (Fig. 4G, lane 3 versus 2 and 4). Together, these results suggested that caspase-mediated cleavage could convert YTHDF2 from an antiviral restriction factor to several proviral fragments (Fig. 4H).

### YTHDF2 regulates viral and cellular gene stability to promote viral replication.

It is known that YTHDF2 binds to m^6^A-modified viral and cellular RNAs to control their stability (11, 16, 29, 45). By m^6^A RNA immunoprecipitation (RIP) followed by reverse transcription-quantitative real-time PCR (RT-qPCR) analysis, we found that EBV immediate early (*ZTA/BZLF1* and *RTA/BRLF1*) and early (*BGLF4*) transcripts are modified by m^6^A, not only revealing the new m^6^A target *BGLF4* but also confirming the results for *ZTA and RTA* captured by m^6^A sequencing (m^6^A-seq) analyses (25) (Fig. S4A). We further demonstrated that YTHDF2 strongly binds to *ZTA*, *RTA*, and *BGLF4* transcripts, given the results from YTHDF2 RIP coupled with RT-qPCR analysis (Fig. S4B). To demonstrate whether YTHDF2 regulates the decay of EBV lytic gene transcripts, we monitored *ZTA* and *RTA* degradation after actinomycin D treatment of control or YTHDF2-depleted Akata (EBV^+^) cells preinduced with anti-IgG for 24 h. We found that the half-lives of both *ZTA* and *RTA* are significantly increased when YTHDF2 is knocked out (Fig. S4C and D). Together, all these results suggested that YTHDF2 binds to EBV lytic transcripts through m^6^A modifications (Fig. S4) and then promotes their decay to restrict EBV reactivation (Fig. 2C and E).

**FIG 5:**
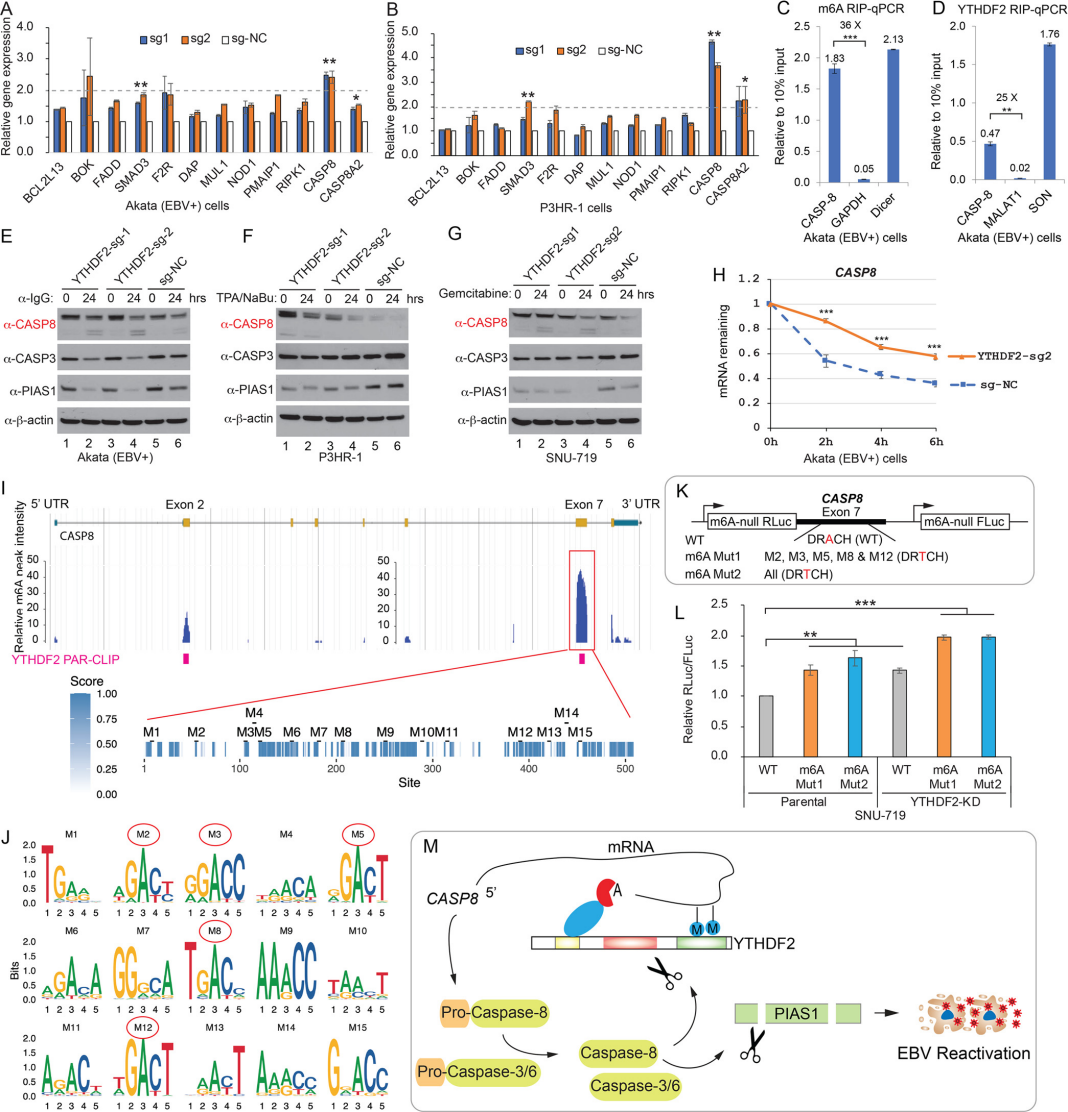
YTHDF2 regulates *CASP8* mRNA stability through m^6^A modifications. (A and B) YTHDF2 depletion promotes *CASP8* mRNA expression. Akata (EBV^+^) cells and P3HR-1 cells carrying different sgRNAs targeting YTHDF2 (sg1 and sg2) or control (sg-NC) were used to extract total RNA, and qPCR analyses were performed with a group of YTHDF2-targeted cellular genes involved in caspase activation. The values were normalized to the non-YTHDF2 target *HPRT1*. The values for sg-NC were set as 1. (C and D) *CASP8* is modified by m^6^A and YTHDF2 binds to *CASP8*. Akata (EBV^+^) cells were used to perform m6A RIP-qPCR (C) and YTHDF2 RIP-qPCR (D), respectively. Values are displayed as fold change over 10% input. (E to G) YTHDF2 depletion promotes caspase-8 protein expression and PIAS1 cleavage upon lytic induction. Akata (EBV^+^) cells (E), P3HR-1 cells (F), and SNU-719 cells (G) carrying different sgRNAs targeting YTHDF2 or control (sg-NC) were lytically induced by anti-IgG, TPA, and sodium butyrate (NaBu) and gemcitabine treatment for 24 h. Protein expression was monitored by Western blotting using the indicated antibodies. (H) Residual mRNA level of *CASP8* after termination of transcription in control (sg-NC) of YTHDF2-depleted (sg2) Akata (EBV^+^) cells. The cells were induced for lytic induction for 24 h and then treated with actinomycin D. The mRNA levels were analyzed by qRT-PCR. The relative mRNA level at 0 h after actinomycin D treatment was set as 1. (I) *CASP8* m^6^A peaks were extracted from MeT-DB V2.0 database. YTHDF2-PAR-CLIP data were retrieved from reference 16. The exon 7 of *CASP8* with highest m^6^A peaks was analyzed for conservation among sequences derived from 100 vertebrate species. Fifteen potential m^6^A motifs (M1 to M15) were extracted based on the m^6^A consensus motif DRACH. (J) Motif logos were generated for 15 individual sites. Red circles denote highly conserved motifs (M2, M3, M5, M8, and M12) across 100 vertebrate species. (K and L) WT and mutant *CASP8*-exon 7 were cloned into the m^6^A-null *Renilla* luciferase (RLuc) reporter (3′-UTR region), which also expresses firefly luciferase (FLuc) from a separate promoter (K). These three reporter plasmids were transfected into parental or YTHDF2-depleted (YTHDF2 KD) SNU719 cells. The ratio of *Renilla* to firefly luciferase activity (RLuc/FLuc) was calculated (L). The value for the WT in parental cells was set as 1. (M) Model illustrating YTHDF2 regulation of *CASP8* mRNA and caspase-8 regulation of YTHDF2 and PIAS1 in EBV reactivation. Results from three biological replicates are presented. Error bars indicate SD. *, *P*< 0.05; **, *P*< 0.01; ***, *P*< 0.001.

10.1128/mBio.01706-21.4 FIG S4 YTHDF2 binds to EBV transcripts and viral RNAs contain m^6^A modifications. See also Fig. 5. Akata (EBV^+^) cells were lytically induced by IgG cross-linking for 24 h. (A) Total RNA was subjected to m^6^A RIP, followed by RT-qPCR using the indicated primers. Values are fold change over 10% input. GAPDH and Dicer are cellular negative and positive controls, respectively. (B) Cell lysate was collected to detect YTHDF2 binding of viral RNAs by RIP-qPCR. Values are fold change over 10% input. MALAT1 and SON are cellular negative and positive controls, respectively. (C and D) Residual mRNA levels of ZTA and RTA after termination of transcription in control (sg-NC) of YTHDF2-depleted (sg2) Akata (EBV^+^) cells. The cells were induced for lytic induction for 24 h and then treated with actinomycin D. The mRNA levels were analyzed by qRT-PCR. The relative mRNA level at 0 h after actinomycin D treatment was set as 1. Results from three biological replicates are presented. Error bars indicate SD. *, *P*< 0.05; **, *P*< 0.01; ***, *P*< 0.001. Download FIG S4, TIF file, 0.8 MB. Copyright © 2021 Zhang et al. 2021 Zhang et al. https://creativecommons.org/licenses/by/4.0/ This content is distributed under the terms of the Creative Commons Attribution 4.0 International license.

In addition to regulating viral mRNA stability, YTHDF2 was reported to modulate m^6^A-modified cellular transcripts to affect a variety of cellular processes. YTHDF2 targets were identified by YTHDF2 RIP and PAR-CLIP (photoactivatable ribonucleoside cross-linking and immunoprecipitation) data sets from previous publications (16, 46). We found a group of YTHDF2 target genes involved in the biological process “activation of cysteine-type endopeptidase activity involved in apoptotic process,” also known as caspase activation (Fig. S5A). The majority of these proteins have protein-protein interactions with caspase-8 (CASP8). As caspase activation plays a critical role in promoting EBV replication (Fig. 3K) (7), we reasoned that YTHDF2 may regulate these genes to limit viral replication. The RT-qPCR results revealed that the mRNA levels of many potential YTHDF2 targets were elevated after YTHDF2 was knocked out by CRISPR/Cas9. In particular, *CASP8* (encoding caspase-8) had a >2-fold significant increase in both Akata (EBV^+^) and P3HR-1 cells (Fig. 5A and B).

10.1128/mBio.01706-21.5 FIG S5 YTHDF2 cleavage or depletion promotes *CASP8* expression and caspase-8 inhibition limits EBV replication in YTHDF2-depleted cells. See also Fig. 4 and 5. (A) A group of genes in the category “activation of cysteine-type endopeptidase activity involved in apoptotic process” (also called caspase activation) were extracted from YTHDF2 target genes derived from YTHDF2 RIP-seq and PAR-CLIP-seq datasets (16, 49, 50). (B and C) YTHDF2 reconstitution suppresses caspase-8 expression and subsequent caspase activation. Akata (EBV+) YTHDF2-sg2 cells carrying vector control, WT or cleavage-resistant YTHDF2 (D166A/D367A) were created using lentiviral transduction. Western blot analysis showing the levels for caspase-8 (CASP8), cleaved caspase-8, and cleaved caspase substrates (CASP sub.) in these cell lines upon IgG cross-linking (B). *CASP8* mRNA levels were analyzed by RT-qPCR using *CASP8* primers (C). The value for the vector control at 0 h was set as 1. (D and E) Caspase-8 inhibition suppress EBV replication in YTHDF2-depleted cells. Control and YTHDF2-depleted Akata (EBV^+^) cells were either untreated or pretreated with caspase-8 inhibitor (Z-IETD-FMK; 50 μM) for 1 h, and then anti-IgG antibody for 0 to 48 h as indicated. Western blot showing the protein levels of EBV ZTA and RTA and cellular PIAS1 as indicated (D). Extracellular viral DNA was measured by qPCR using primers specific to BALF5 (E). The value for the vector control at 0 h was set as 1. (F and G) Caspase-8 depletion suppresses EBV replication in YTHDF2-depleted cells. YTHDF2-depleted Akata (EBV^+^) cells carrying control sgRNA or CASP8-sg1 were treated with anti-IgG antibody for 0 to 48 h as indicated. Western blot showing the protein levels of EBV ZTA and RTA and cellular PIAS1 as indicated (F). Extracellular viral DNA was measured by qPCR using primers specific to BALF5 (G). The value for the vector control at 0 h was set as 1. Results from three biological replicates are presented. Error bars indicate SD. **, *P* < 0.01; ***, *P* < 0.001. Download FIG S5, TIF file, 1.1 MB. Copyright © 2021 Zhang et al. 2021 Zhang et al. https://creativecommons.org/licenses/by/4.0/ This content is distributed under the terms of the Creative Commons Attribution 4.0 International license.

To test whether *CASP8* mRNA is modified by m^6^A in EBV-positive cells, we performed m^6^A RIP followed by RT-qPCR using Akata (EBV^+^) cells. The results demonstrated that *CASP8* mRNA is strongly modified by m^6^A, compared to the controls (Fig. 5C). In addition, YTHDF2 indeed bound to *CASP8* mRNA, as revealed by YTHDF2 RIP-qPCR analysis (Fig. 5D). Consistent with mRNA elevation, caspase-8 protein level was also increased upon YTHDF2 depletion (Fig. 5E to G, lanes 1 and 3 versus 5). Upon lytic induction, we observed enhanced caspase-8 activation and consequently PIAS1 degradation (Fig. 5E to G, lanes 2 and 4 versus 6). Conversely, WT and D166A/D367A mutant YTHDF2 reconstitutions led to reduced caspase-8 protein levels and caspase activation upon lytic induction, with the D166A/D367A mutant having the strongest effects (Fig. S5B, lanes 1 to 3 versus 4 to 6 and7 to 9). By RT-qPCR analysis, we further found that *CASP8* mRNA level was increased in cells expressing WT YTHDF2 but not the D166A/D367A mutant following lytic induction (Fig. S5C). To understand the relationship between YTHDF2 regulation of caspase-8 and EBV replication, we pretreated the YTHDF2-depleted Akata (EBV^+^) cells with or without a caspase-8 inhibitor and then induced the lytic cycle by anti-IgG treatment. We found that caspase-8 inhibition reduced EBV ZTA and RTA expression and consequently inhibited EBV virion production even when YTHDF2 was depleted (Fig. S5D and E, lane 7 versus 9, lane 8 versus 10, lane 12 versus 14, and lane 13 versus 15). To further confirm our results, we created a *CASP8/YTHDF2* double-knockout cell line using Akata (EBV^+^) cells. We found that, compared with *YTHDF2* knockout cells, both EBV lytic protein expression and viral copy numbers were significantly reduced in *CASP8/YTHDF2* double-knockout cells (Fig. S5F, lanes 2 and 3 versus 5 and 6, and Fig. S5G). To demonstrate whether YTHDF2 regulates the decay of *CASP8* transcript, we monitored *CASP8* mRNA degradation after actinomycin D treatment. We found that the half-life of *CASP8* is significantly increased in YTHDF2-depleted cells (Fig. 5H).

Together, our results suggested that the depletion of YTHDF2 can promote EBV replication partially through enhanced caspase-8 activation. m^6^A modifications are mainly located near the stop codon region to regulate mRNA decay (16, 47, 48). By mining the m^6^A modification database (49, 50) and YTHDF2-PAR-CLIP data sets (16), we found that *CASP8-*exon 7 has the highest m^6^A peaks that are closed to the stop codon (Fig. 5I). Using the conserved m^6^A motif DRACH (D = G, A, or U; R = G or A; H = A, U, or C) (51), we identified 15 potential m^6^A sites that are evenly distributed across the entirety of exon 7 (Fig. 5I). We found that motif 2 (M2), M3, M5, M8 and M12 are highly conserved across more than 90 vertebrates, including humans, mice, bats, and zebrafish, while the others likely evolved gradually during evolution (Fig. 5J and Table S1C).

10.1128/mBio.01706-21.10 TABLE S1 (A) Primers, templates, and key resources used in this study. (B) Caspase cleavage motif sequences (corresponding to cleavage sites in human YTHDF2, YTHDF1, YTHDF3, and WTAP) extracted from vertebrate species. Related to Fig. S2, S7, and S8. (C) m^6^A motif sequences (corresponding to putative m^6^A motifs in human *CASP8*-Exon 7) extracted from vertebrate species. Related to Fig. 5. Download Table S1, XLSX file, 0.06 MB. Copyright © 2021 Zhang et al. 2021 Zhang et al. https://creativecommons.org/licenses/by/4.0/ This content is distributed under the terms of the Creative Commons Attribution 4.0 International license.

**FIG 6:**
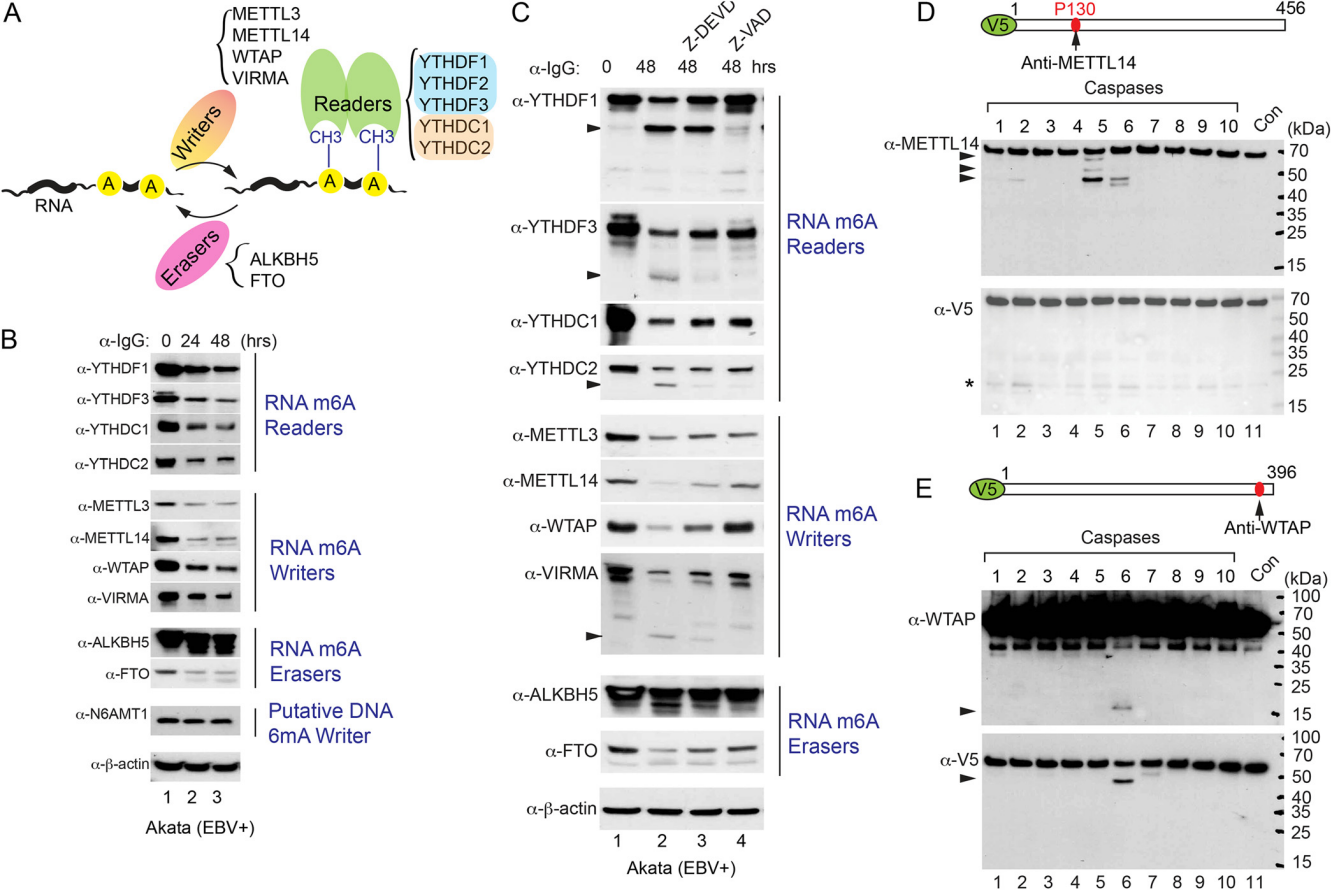
Caspases cleave m^6^A RNA modification pathway proteins. (A) Diagram summarizing the major writers, readers, and erasers involved in the m^6^A RNA modification pathway. (B) Downregulation of m^6^A RNA modification pathway proteins during EBV reactivation. Akata (EBV^+^) cells were treated with anti-IgG antibody to induce EBV reactivation for 0, 24, and 48 h. Western blotting was performed using antibodies as indicated. N6AMT1 and β-actin blots were included as controls. (C) Caspase inhibition blocks the degradation of m^6^A RNA modification pathway proteins. The Akata (EBV^+^) cells were either untreated or pretreated with a caspase-3/-7 inhibitor (Z-DEVD-FMK; 50 μM) or a pan-caspase inhibitor (Z-VAD-FMK; 50 μM) for 1 h, and then anti-IgG antibody was added for 48 h. Western blotting was performed using antibodies as indicated. (D and E) V5-METTL14 (D) and V5-WTAP (E) were incubated with individual caspase for 2 h at 37°C. Western blotting was performed using anti-METTL14, anti-V5, and anti-WTAP antibodies as indicated. The locations of antibody recognition epitopes are labeled. Arrowheads indicate cleaved fragments; the star indicates nonspecific bands.

10.1128/mBio.01706-21.7 FIG S7 The conservation of YTHDF2, YTHDF1, and YTHDf3 cleavage sites. See also Fig. 6 and Table S1B. (A) Sequence alignment of YTHDF2 with YTHDF1 and YTHDF3 using the constraint-based multiple alignment tool (COBALT). The corresponding cleavage motifs are highlighted by green boxes. (B) Sequence alignment of YTHDF2 sequences from 10 representative species using COBALT. The cleavage motifs are highlighted in yellow. (C) Motif analysis showing the conservation of YTHDF1-D164/YTHDF3-D168 and the surrounding amino acids. Amino acid sequences were extracted from 97 vertebrate species, and motif logos were generated using WebLogo. Download FIG S7, TIF file, 3.0 MB. Copyright © 2021 Zhang et al. 2021 Zhang et al. https://creativecommons.org/licenses/by/4.0/ This content is distributed under the terms of the Creative Commons Attribution 4.0 International license.

**FIG 7:**
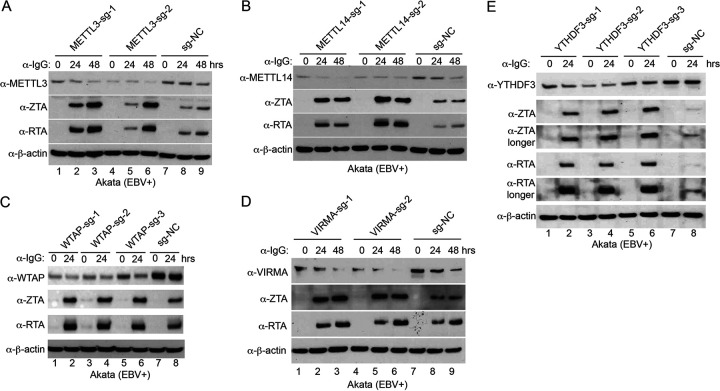
Depletion of m^6^A writers and reader YTHDF3 promotes EBV lytic replication. Akata (EBV^+^) cells were used to establish stable cell lines using 2 or 3 different guide RNA constructs targeting METTL3 (A), METTL14 (B), WTAP (C), VIRMA (D), and YTHDF3 (E) and a nontargeting control (sg-NC). The cells were untreated or lytically induced with anti-IgG-mediated BCR activation. Cellular and viral protein expression levels were monitored by Western blotting using the indicated antibodies.

10.1128/mBio.01706-21.8 FIG S8 The role of m^6^A writers, readers, and erasers in EBV replication. See also Fig. 6 and 7 and Table S1B. (A) METTL3 is weakly cleaved by caspases. V5-METTL3 was incubated with individual caspase for 2 h at 37°C. Western blotting was performed using anti-METTL3 and anti-V5 antibodies as indicated. The locations of antibody recognition epitopes were labeled as indicated. The positions of weakly cleaved fragments are indicated by arrowheads. The star denotes nonspecific bands. (B) WTAP is cleaved at D302. V5-tagged WTAP D301A/D302A and D301A mutants were incubated with individual recombinant caspase for 2 h. Western blotting was performed using the indicated antibodies. Arrowheads indicate cleaved fragments. (C) Sequence alignment of WTAP sequences from 10 representative species using the constraint-based multiple alignment tool (COBALT). The cleavage motifs are highlighted in yellow. (D) Motif analysis showing the conservation of WTAP D302 and the surrounding amino acids. Amino acid sequences were extracted from 97 vertebrate species, and motif logos were generated using WebLogo. (E and F) Depletion of YTHDF1 or ALKBH5 does not affect EBV protein expression. Akata (EBV^+^) cells were used to establish stable cell lines using 2 different guide RNA constructs targeting YTHDF1 (D) and ALKBH5 (E) and a nontargeting control (sg-NC). The cells were untreated or lytically induced with anti-IgG-mediated BCR activation. Cellular and viral protein expression levels were monitored by Western blotting using the indicated antibodies. (G to P) Depletion of m^6^A writers and reader YTHDF3 promotes EBV lytic replication. Akata (EBV^+^) cells were used to establish stable cell lines using 2 or 3 different guide RNA constructs targeting METTL3 (G and H), METTL14 (I and J), WTAP (K and L), VIRMA (M and N), and YTHDF3 (O and P) and a nontargeting control (sg-NC). The cells were untreated or lytically induced with anti-IgG-mediated BCR activation for 24 or 48 h. EBV *ZTA* and *RTA* mRNA expression levels were monitored by RT-qPCR (G, I, K, M, and O). Extracellular viral DNA was measured by qPCR using primers specific to BALF5 (H, J, L, N, and P). The value for the vector control at 0 h was set as 1. Results from three biological replicates are presented. Error bars indicate SD. **, *P* < 0.01; ***, *P* < 0.001. Download FIG S8, TIF file, 2.2 MB. Copyright © 2021 Zhang et al. 2021 Zhang et al. https://creativecommons.org/licenses/by/4.0/ This content is distributed under the terms of the Creative Commons Attribution 4.0 International license.

To determine whether m^6^A modifications contribute to *CASP8* mRNA stability, we synthesized the exon 7 DNA of WT *CASP8* (without the first 20 bp) and its mutant counterparts with conserved motifs (M2, M3, M5, M8, and M12) or all putative motifs mutated (Fig. 5K). Luciferase reporters were created with WT or mutant DNA inserted into the 3′-untranslated region (3′-UTR) of an m^6^A-null vector (Fig. 5K). These reporters were modified to silently mutate all putative m^6^A sites in the *Renilla* and firefly luciferase genes to specifically test the function of m^6^A modifications on *CASP8-*exon 7 (52). We then transfected these plasmids individually into SNU-719 cells carrying YTHDF2 or having YTHDF2 depleted by CRISPR/Cas9. Disruption of m^6^A modification by mutations enhanced the relative luciferase activity compared to the WT reporter (Fig. 5L). In addition, depletion of YTHDF2 also enhanced the relative luciferase activity (Fig. 5L), suggesting that YTHDF2 directly regulates *CASP8* stability. We also noticed a further enhancement of relative luciferase activity for mutant reporters in YTHDF2-depleted cells (Fig. 5L), suggesting regulation of *CASP8* stability by additional m^6^A readers, e.g., YTHDF1 or YTHDF3 (20).

Together, these results suggested that YTHDF2 depletion by CRISPR/Cas9 or its cleavage by caspases could promote EBV replication by upregulating caspase-8, which further enhances the cleavage of antiviral restriction factors, including PIAS1 and YTHDF2 (Fig. 5M) (7).

### m^6^A pathway proteins are regulated by caspase-mediated cleavage.

The m^6^A RNA modification machinery contains writers, readers, and erasers that mediate methylation, RNA binding, and demethylation steps, respectively (Fig. 6A). Prompted by our YTHDF2 results, we predicted that other members involved in the m^6^A pathway may be downregulated by caspase-mediated cleavage during viral replication. To evaluate this possibility, we first monitored the protein levels of additional m^6^A readers (YTHDF1, YTHDF3, YTHDC1, and YTHDC2), m^6^A writers (METTL3, METTL14, VIRMA, and WTAP), and m^6^A erasers (ALKBH5 and FTO). Interestingly, except ALKBH5, all proteins were significantly downregulated upon lytic induction (Fig. 6B; Fig. S6A to K). As a control, the protein level of a putative DNA *N*^6^-methyladenine (6mA) writer, N6AMT1, did not change upon lytic induction (Fig. 6B; Fig. S6L), suggesting a specific regulation of the m^6^A RNA modification pathway by cellular caspases.

10.1128/mBio.01706-21.6 FIG S6 m^6^A readers, writers and erasers are cleaved upon lytic induction. See also Fig. 6. Full blots for Fig. 6B showing the generation of cleaved fragments for m^6^A readers, writers and erasers. Western blotting was performed using antibodies as indicated. The N6AMT1 blot was included as a control. Download FIG S6, TIF file, 1.4 MB. Copyright © 2021 Zhang et al. 2021 Zhang et al. https://creativecommons.org/licenses/by/4.0/ This content is distributed under the terms of the Creative Commons Attribution 4.0 International license.

To further demonstrate the role of caspases in the downregulation of m^6^A pathway proteins, we examined their protein levels in the presence of caspase-3/7 or pan-caspase inhibitors. Indeed, we found that caspase inhibition could restore the protein levels of YTHDF1, YTHDF3, METTL14, WTAP, VIRMA, and FTO (Fig. 6C). The partial restoration of YTHDC1, YTHDC2, and METTL3 suggested that these proteins are controlled partially by caspase cleavage and partially by other protein degradation mechanisms (Fig. 6C).

YTHDF2 shares high sequence homology with YTHDF1 and YTHDF3. Sequence alignment showed that the cleavage motif AMID*G, but not NGVD*G, is partially conserved among YTHDF-family proteins (Fig. S7A). Because TVVD*G in YTHDF1 and AITD*G in YTHDF3 are also evolutionarily conserved (Fig. S7B and C), we reasoned that YTHDF1 and YTHDF3 are subjected to caspase-mediated cleavage on the N-terminal sites. Consistent with our prediction, not only did YTHDF1 and YTHDF3 protein levels decrease, but also, a cleaved band near 50 kDa was generated upon lytic induction by IgG-cross-linking, matching the calculated molecular weights of C-terminal fragments generated by cleavage on the N-terminal sites (Fig. S6B and C). In addition, cleaved fragments were detected for YTHDC1, YTHDC2, METTL14, VIRMA, and FTO (Fig. S6D to K).

To further demonstrate that the m^6^A writers are cleaved by caspase, we performed *in vitro* cleavage assay using purified proteins. Interestingly, we found that METTL14 is mainly cleaved by caspase-2, -5, and -6, revealed by anti-METTL14 antibody (Fig. 6D), whereas WTAP is mainly cleaved by caspase-6 (Fig. 6E). However, we observed only trace amounts of METTL3 cleavage by caspase-4, -5, -6, and -7, revealed by anti-METTL3 antibody (Fig. S8A).

The cleavage patterns suggested that METTL14 is cleaved at multiple sites, while WTAP is possibly cleaved at one major site near the C terminus (Fig. 6E). Based on the C-terminal fragment molecular weight (15 kDa), we reasoned that D301 or D302 is cleaved. To examine this, we generated WTAP mutants (D301A and D301A/D302A) and performed *in vitro* cleavage experiments. Interestingly, only the D301A/D302A mutant could block WTAP cleavage (Fig. S8B), indicating that D302 is the major cleavage site. Sequence analysis revealed that the core cleavage motif TEDD*F is evolutionarily conserved (Fig. S8C). Compared to cleavage sites normally followed by glycine, serine, or alanine (36), the discovery of a conserved phenylalanine after the cleavage site extends our knowledge of substrate recognition by caspase (Fig. S8D).

### Depletion of m^6^A writers (METTL3, METTL14, WTAP, and VIRMA) and the reader YTHDF3 promotes EBV replication.

The aforementioned studies suggest that YTHDF2 restricts viral replication through m^6^A modifications. We reasoned that the disruption of the m^6^A writer complex and other readers might also promote viral replication. To test our hypothesis, we used a CRISPR/Cas9 genomic editing approach to deplete METTL3, METTL14, VIRMA, and WTAP in Akata (EBV^+^) cells. Consistent with our prediction, depletion of METTL3, METTL14, VIRMA, and WTAP all facilitated the expression of EBV ZTA and RTA (Fig. 7A and B; Fig. 7D, lanes 2, 3, 5, and 6 versus 8 and 9; Fig. 7C, lanes 2, 4, and 6 versus 8). Because YTHDF1 and YTHDF3 share a redundant role with YTHDF2, we knocked out these two genes using CRISPR/Cas9 genomic editing approaches. Interestingly, depletion of YTHDF3 rather than YTHDF1 promoted EBV ZTA and RTA expression (Fig. 7E; Fig. S8E). In addition, depletion of the major m^6^A eraser ALKBH5 did not affect EBV protein accumulation (Fig. S8F).

To further confirm the key roles of m^6^A writers and readers in EBV gene expression and life cycle, we measured *ZTA* and *RTA* gene expression by RT-qPCR and virion-associated DNA copy numbers by qPCR for cells with individual gene depleted by CRISPR/Cas9. We found that the depletion of METTL3, METTL14, WTAP, VIRMA, and YTHDF3 all promoted viral gene expression (Fig. S8G, I, K, M, and O) and facilitated viral replication upon lytic induction (Fig. S8H, J, L, N, and P).

All results together suggested that, in addition to m^6^A readers, disruption of the m^6^A writer complex by caspases further fosters EBV reactivation upon lytic induction.

## DISCUSSION

**FIG 8:**
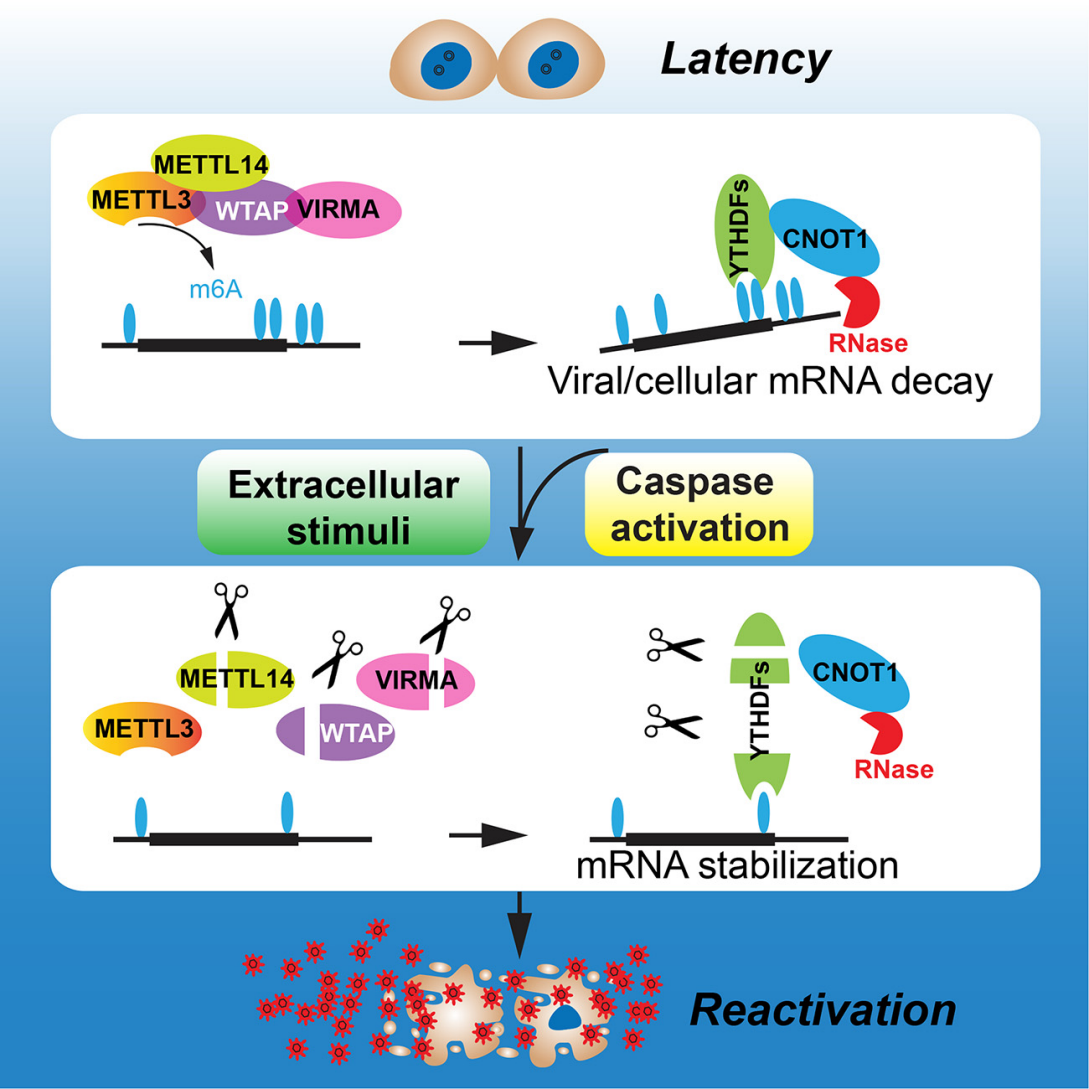
Model of how caspase-mediated cleavage of m^6^A pathway writers and readers facilitates EBV reactivation. During latency, m^6^A writers deposit the methyl group onto key viral and cellular mRNAs, which are subsequently destabilized by m^6^A readers. Upon reactivation, cellular caspases are activated. On one hand, caspases cleave the writers to limit the m^6^A modification process, and on the other hand, caspases cleave the readers to limit RNA decay by the CNOT-CCR4 complex, which together foster EBV reactivation and drive the production of massive amounts of viruses.

Caspase activation and the m^6^A RNA modification pathway have documented essential roles in viral infections. However, it is unknown whether caspases regulate any members of the m^6^A machinery to foster viral infection. Our study established an elegant regulation model involving the regulation of multiple members of the m^6^A RNA modification pathway by caspase-mediated cleavage, which plays a crucial role in regulating the reactivation of EBV (Fig. 8).

The m^6^A reader YTHDF2 has been shown to regulate the life cycle of diverse viruses, including EBV and KSHV (25, 28, 29). However, the mechanism by which YTHDF2 is regulated during viral replication has not been determined. Based on motif searching and further validation, we found that YTHDF2 was cleaved by caspases on D166 and D367. Both cleavage sites, especially D166, are evolutionarily conserved across a diverse group of vertebrates ranging from human to zebrafish, highlighting that maintaining these cleavage sites during evolution is important for normal cellular processes.

In addition to YTH domain binding to m^6^A, YTHDF2 contains a low-complexity domain with disorder-promoting amino acids in the N-terminal and central parts. Our 3D structure modeling on full-length YTHDF2 clearly shows two cleavage sites on the surface area with flexible turns. Because the low-complexity domain is responsible for YTHDF2-mediated phase separation to form liquid droplets with high concentrations of protein and RNA (21–23), our predicted protein structure model will provide valuable insights into the regulation of YTHDF family proteins under normal conditions.

Although YTHDF2 regulation of viral transcript stability contributes to viral replication (25, 28, 29), the extent to which cellular gene regulation by YTHDF2 participates in this process has been largely unexplored. By analyzing YTHDF2 targets transcripts, we identified *CASP8* as a putative YTHDF2 target involved in EBV replication. We demonstrated that the *CASP8* transcript is modified by m^6^A and bound by YTHDF2 and that depletion of YTHDF2 promotes *CASP8* mRNA level, caspase-8 protein level, and hence its activation and subsequent cleavage of PIAS1 favoring EBV lytic replication.

Intriguingly, we found 15 putative m^6^A sites in the second-to-last exon. Among them, 5 sites are conserved across a diverse group of species. Although DNA sequences differ significantly across species, our discovery of conserved m^6^A sites in regulating *CASP8* stability highlights the importance of m^6^A RNA modification in controlling the basal levels of key cellular genes during evolution.

Importantly, our work on YTHDF2 led to the discovery of multiple m^6^A RNA modification pathway proteins as caspase substrates and EBV restriction factors. The extension of YTHDF2 to YTHDF1 and YTHDF3 as caspase substrates was readily revealed by sequence alignment and cleavage pattern analyses. Interestingly, WTAP is predominately cleaved by caspase-6 on D302 within an evolutionarily conserved motif that differs significantly from canonical cleavage motifs. The cleavage pattern of METTL14 suggested that multiple sites are cleaved, possibly including D29 (EASD*S), revealed by a large-scale proteomics study (53). It will be interesting to identify all cleavage sites for other m^6^A pathway proteins and examine how specific cleavage contributes to EBV latency and lytic replication in the future. Importantly, the discovery of new cleavage motifs would also guide us to discover novel caspase substrates using the motif searching method and to determine the biological function of protein cleavage in viral life cycle and normal cellular processes.

Emerging studies suggested that mRNA decay pathways play an important role in restricting gamma-herpesvirus reactivation. Proteins involved in nonsense-mediated mRNA decay have been shown by the Gack and Karijolich labs to restrict EBV and KSHV replication (54, 55). Our current discovery of multiple proteins within the m^6^A RNA modification pathway as EBV restriction factors further enhances our understanding of the control of herpesvirus reactivation at the RNA level.

EBV infection of primary human B cells gradually establishes latency by reprogramming the cellular environment. Considering the role of YTHDF2 in controlling EBV latency, we also extracted the RNA- and protein-level data from the transcriptomic and proteomic analyses of EBV infection of primary human B cells by the Hammerschmidt group and the Gewurz group (56, 57), respectively. Interestingly, we found that EBV infection led to enhanced YTHDF2 RNA and protein expression and reduced CASP8 expression (Fig. S9). There were also a transient increase and then gradual decrease of EBV latent and lytic gene expression, suggesting that YTHDF2 may also control their expression during latency establishment (Fig. S9).

10.1128/mBio.01706-21.9 FIG S9 The expression of YTHDF2 negatively correlates with the mRNA and protein expression of CASP8, EBV EBNA2, and BLRF2 upon EBV infection of primary human B cells. The RNA (A) and protein (B) levels of YTHDF2, CASP8, EBV EBNA2, and BRLF2 were analyzed for EBV infection of primary human B cells. The data were extracted from the transcriptomic (http://ebv-b.helmholtz-muenchen.de/) (57) and proteomic (56) analyses, respectively. Download FIG S9, TIF file, 0.5 MB. Copyright © 2021 Zhang et al. 2021 Zhang et al. https://creativecommons.org/licenses/by/4.0/ This content is distributed under the terms of the Creative Commons Attribution 4.0 International license.

There are several limitations to our study. For example, overexpression of individual fragment may not represent the real cleavage situation and therefore may lead to the enhanced phenotypes that we observed. The existence of several fragments in real cleavage situations may be coordinated to promote viral replication. In addition to *CASP8*, the expression of epigenetic regulators, including histone modifiers and transcription factors, may be altered upon the depletion of YTHDF2 and other m^6^A pathway genes (58). Indeed, the transcripts of histone acetyltransferases p300/CBP and histone methyltransferase EZH2 have been shown to be regulated by m^6^A modifications (59, 60). All these factors have been implicated in gammaherpesvirus latency and reactivation (61–63). The manipulation of m^6^A pathway regulators may also change chromatin regulatory RNAs (64). The chromatin regulatory RNAs may also control the viral and cellular chromatin status important for EBV latency and/or reactivation. METTL3 and YTHDC1 were reported to regulate heterochromatin formation via RNA m^6^A methylation in mouse embryonic stem cells (65). The cross talk between epitranscriptomics and epigenetics in herpesvirus latency and lytic replication is an exciting area to be explored in the future.

In summary, our work has illustrated the value of a motif-based searching approach for the discovery of novel caspase substrates that are critical for viral replication. We have uncovered a unique caspase regulation mechanism for the m^6^A RNA modification pathway, which is essential for the replication of a ubiquitous tumor virus. The discovery of conserved caspase cleavage motifs will guide us to discover novel caspase substrates with broad biological significance, provide valuable insights into the regulation of viral life cycle, and illuminate the key molecular mechanisms controlling normal cellular processes and disease progression.

## MATERIALS AND METHODS

### Cell lines and cultures.

Akata (EBV^+^), Akata-4E3 (EBV^−^), P3HR-1, and SNU-719 cells were cultured in RPMI 1640 medium supplemented with 10% fetal bovine serum (FBS) (catalog no. 26140079; Thermo Fisher Scientific) in 5% CO_2_ at 37°C. 293T cells were cultured in Dulbecco’s modified Eagle medium (DMEM) supplemented with 10% FBS (catalog no. 26140079; Thermo Fisher Scientific) in 5% CO_2_ at 37°C. All cell line information is listed in Table S1A.

### Plasmid construction.

Halo-V5-YTHDF2 (full-length and truncation mutants), Halo-V5-METTL3, Halo-V5-METTL14, and Halo-V5-WTAP were cloned into the pHTN HaloTag CMV-neo vector (catalog no. G7721; Promega) using Gibson assembly methods as previously described (7, 38). The Halo-V5-YTHDF2 mutant (D166A/D367A) and Halo-V5-WTAP mutants (D301A and D301A/D302A) were generated using the QuikChange II site-directed mutagenesis kit (Stratagene) following the manufacturer’s instructions. All primers are listed in Table S1A.

### Target gene depletion by CRISPR/Cas9 genome editing.

To deplete YTHDF2, EIF4H, MAGEA10, SORT1, MTA1, EHMT2, METTL3, METTL14, WTAP, VIRMA, YTHDF1, YTHDF3, and ALKBH5, two or three different single guide RNAs (sgRNAs) were designed and cloned into lentiCRISPR v2-Puro vector (a gift from Feng Zhang; Addgene plasmid no. 52961) (66) or lentiCRISPR v2-BLAST vector (a gift from Mohan Babu; Addgene plasmid no. 83480) or lentiCRISPR v2-Hygro vector (a gift from Joshua Mendell, Addgene plasmid no. 91977) (67). Packaging 293T cells were transfected with targeted gene sgRNAs or negative-control vector (nontargeting sg-NC) and helper vectors (pMD2.G and psPAX2; gifts from Didier Trono; Addgene plasmid no. 12259 and 12260, received via Yue Sun) using Lipofectamine 2000 reagent (catalog no. 11668019; Life Technologies). Medium containing lentiviral particles and 8 mg/ml Polybrene (Sigma-Aldrich, St. Louis) was used to infect Akata (EBV^+^) cells, P3HR-1 cells, and SNU-719 cells. The stable cell lines were selected and maintained in RPMI medium supplemented with 2 μg/ml puromycin, 100 μg/ml hygromycin, or 10 μg/ml blasticidin.

To knock out three caspases (CASP3, CASP8, and CASP6), first, one sgRNA targeting CASP3 (CASP3-sg1) was cloned into the lentiCRISPR v2-Puro vector, and Akata (EBV^+^) cells were used to create a CASP3-depleted cell line, Akata (EBV^+^)-CASP3-sg1, by CRISPR/Cas9 (puromycin resistant). Second, one sgRNA targeting CASP8 (CASP8-sg1) and one control sgRNA (sg-NC) were cloned into lentiCRISPR v2-BLAST vector. The constructs were packaged, and lentiviral particles were used to infect Akata (EBV^+^)-CASP3-sg1 and control Akata (EBV^+^)-sg-NC cells to generate Akata (EBV^+^)-CASP3-sg1/CASP8-sg1 and Akata (EBV^+^)-sg-NC/sg-NC cell lines. Third, two different sgRNAs targeting CASP6 (CASP6-sg1 and CASP6-sg2) and one control sgRNA (sg-NC) were designed and cloned into the lentiCRISPR v2-Hygro vector. The constructs were packaged, and lentiviral particles were used to infect Akata (EBV^+^)-CASP3-sg1/CASP8-sg1 and control Akata (EBV^+^)-sg-NC/sg-NC cells. The established Akata (EBV^+^)-CASP3-sg1/CASP8-sg1/CASP6-sg1, Akata (EBV^+^)-CASP3-sg1/CASP8-sg1/CASP6-sg2, and control Akata (EBV^+^)-sg-NC/sg-NC/sg-NC were maintained in RPMI medium supplemented with 100 μg/ml hygromycin, 2 μg/ml puromycin, and 10 μg/ml blasticidin. The target guide RNA sequences are listed in Table S1A.

Similarly, to create a *YTHDF2/CASP8* double-knockout cell line, first, one sgRNA targeting *CASP8* (CASP8-sg1) and control sgRNA in lentiCRISPR v2-BLAST vector were used to create *CASP8*-knockout and control cell lines [Akata (EBV^+^)-CASP8-sg1 and Akata (EBV^+^)-sg-NC (blasticidin resistant)]. Second, lentiviral particles carrying one sgRNA targeting *YTHDF2* (YTHDF2-sg1; puromycin resistant) were used to infect Akata (EBV^+^)-CASP8-sg1 and control Akata (EBV^+^)-sg-NC cell lines. The established Akata (EBV^+^)-CASP8-sg1/YTHDF2-sg1 and Akata (EBV^+^)-sg-NC/YTHDF2-sg1 cell lines were maintained in RPMI medium supplemented with 2 μg/ml puromycin and 10 μg/ml blasticidin. The target guide RNA sequences are listed in Table S1A.

### Lentiviral transduction of YTHDF2.

The pLenti-C-Myc-DDK-P2A-BSD and pCMV6-Entry-YTHDF2 were purchased from Origene. The specific variants were generated by site-directed mutagenesis in pCMV6-Entry-YTHDF2 using the QuikChange II site-directed mutagenesis kit (catalog no. 200521; Stratagene) according to the manufacturer’s instructions. The truncated YTHDF2 was cloned into pCMV6-Entry vector using Gibson assembly methods as we described previously (7, 38). Subsequently, the WT, mutated, and truncated YTHDF2 in pCMV6-Entry vector were digested using AsiSI and MluI and subcloned into the pLenti-C-Myc-DDK-P2A-BSD vector. To prepare lentiviruses, 293T cells were transfected with lentiviral vector containing the target gene and the helper vectors (pMD2.G and psPAX2) using Lipofectamine 2000 reagent. The supernatants were collected 48 h posttransfection to infect Akata (EBV^+^) cells, and then stable cell lines were selected in RPMI medium containing 10 μg/ml blasticidin.

For YTHDF2 reconstitution, we first transduced the Akata (EBV^+^) cells with lentiviruses carrying vector control, WT and mutant (D166A/D367A) YTHDF2 to establish cell lines in RPMI medium containing 10 μg/ml blasticidin. We then depleted endogenous YTHDF2 by lentiviral transduction of YTHDF2-sg2 into these three cell lines. The obtained stable cell lines were selected in RPMI medium supplemented with 2 μg/ml puromycin and 10 μg/ml blasticidin.

### Lytic induction and cell treatment.

For lytic induction in Akata (EBV^+^) cell lines, the cells were treated with IgG (1:200; catalog no. 55087; MP Biomedicals) for 0 to 48 h. Akata-4E3 (EBV^−^) cells were treated similarly as controls. To induce the EBV lytic cycle in P3HR-1 cells, the cells were triggered with tetradecanoyl phorbol acetate (TPA; 20 ng/ml) and sodium butyrate (3 mM) for 0 to 48 h. For EBV lytic induction in SNU-719 cells, the cells were treated with gemcitabine (1 μg/ml) or TPA (20 ng/ml) and sodium butyrate (3 mM) for 0 to 48 h. For the caspase inhibition assay, Akata (EBV^+^), P3HR-1, and SNU719 cells were untreated or pretreated with caspase inhibitors (50 μM) for 1 h and then treated with lytic inducers for an additional 24 or 48 h. All key reagent information is listed in Table S1A.

### mRNA stability assay.

To measure mRNA stability, Akata (EBV^+^)-YTHDF2-sg2 and sg-NC control cells were seeded in 6-well plates, prelytically induced with anti-IgG for 24 h, and then treated with actinomycin D (5 μg/ml) (catalog no. A1410; Sigma-Aldrich) to inhibit transcription. The cells were collected at 0, 2, 4, and 6 h after treatment. The total RNA was extracted with an Isolate II RNA minikit (Bioline) and analyzed by qRT-PCR with specific primers for *ZTA*, *RTA*, and *CASP8* (Table S1A).

### Cell lysis and immunoblotting.

Cell lysates were prepared in lysis buffer supplemented with protease inhibitors (Roche) as described previously (7). Protein concentration was determined by Bradford assay (Bio-Rad). The proteins were separated on 4 to 20% TGX gels (Bio-Rad) and transferred to polyvinylidene (PVDF) membranes using a semidry transfer system. Membranes were blocked in 5% milk and probed with primary and horseradish peroxidase-conjugated secondary antibodies. All antibody information is listed in Table S1A.

### Protein expression and purification.

Halo-V5-YTHDF2 (WT and D166A/D367A mutant), Halo-V5-METTL3, Halo-V5-METTL14, and Halo-V5-WTAP (WT and D301A and D301/D302A mutants) proteins were expressed and purified as previously described (68). Briefly, Halo-tagged plasmids were transfected into 293T cells at 50 to 60% confluence. Two T175 flasks of transfected cells were harvested 48 h posttransfection at 100% confluence and lysed with 25 ml HaloTag protein purification buffer (50 mM HEPES [pH 7.5], 150 mM NaCl, 1 mM dithiothreitol [DTT], 1 mM EDTA and 0.005% NP-40/IGEPAL CA-630) with protease inhibitor cocktail. Halo-V5-tagged proteins were enriched using the Halo-tag resin, and proteins were eluted from the resin by washing 3 times with 0.5 ml HaloTag protein purification buffer containing 20 μl Halo-TEV (tobacco etch virus) protease. The eluted proteins were stored at −80°C for further use.

### RT-qPCR.

Total RNA was isolated from cells with an Isolate II RNA minikit (Bioline) according to the manufacturer’s instructions. Total RNA was reverse transcribed to cDNA using a high-capacity cDNA reverse transcription kit (Invitrogen). qPCR was performed using Brilliant SYBR green qPCR master mix (Agilent Technology) with specific primers listed in Table S1A. The relative expression of target mRNA was normalized by β-actin gene or *HPRT1* expression level.

### EBV DNA detection.

To measure EBV replication, levels of cell-associated viral DNA and virion-associated DNA were determined by qPCR analysis. For intracellular viral DNA, total genomic DNA was extracted using a genomic DNA purification kit (catalog no. A1120; Promega). For extracellular viral DNA, the supernatant was treated with RQ1 DNase at 37°C for 1 h, which was deactivated by RQ1 DNase stop solution, followed by release of virion-associated DNA with proteinase and SDS treatment as previously described (6). The DNA was then purified by phenol-chloroform–isoamyl alcohol extraction. The relative viral DNA copy numbers were determined by qPCR using primers to the *BALF5* gene. The reference β-actin gene was used for data normalization.

### *In vitro* caspase cleavage assay.

Purified V5-YTHDF2 (WT and mutants), V5-WTAP (WT and mutants), WT V5-METTL3, and WT V5-METTL14 were incubated with individual caspase in caspase assay buffer (50 mM HEPES [pH 7.2], 50 mM NaCl, 0.1% CHAPS {3-[(3-cholamidopropyl)-dimethylammonio]-1-propanesulfonate}, 10 mM EDTA, 5% glycerol, and 10 mM DTT) at 37°C for 2 h with gentle agitation. Reactions were stopped by boiling in 2× SDS sample buffer, and samples were analyzed by Western blotting.

### IP assay.

293T cells (50 to 60% confluence) were cotransfected with Halo-V5 tagged WT or truncated YTHDF2 and HA tagged CNOT1-SH domain using Lipofectamine 2000. The cells were harvested 48 h posttransfection and lysed in radioimmunoprecipitation assay (RIPA) lysis buffer (50 mM Tris-HCl, 150 mM NaCl, 1% NP-40, 1% deoxycholate, 0.1% SDS, and 1 mM EDTA) containing protease inhibitor cocktail (catalog no. 11836153001; Roche) and phosphatase inhibitors (1 mM Na_3_VO_4_ and 1 mM NaF). IP was carried out as previously described (7, 38).

### RNA-binding protein immunoprecipitation.

RNA-binding protein immunoprecipitation (RIP) was performed using a Magna RIP kit (catalog no. 17-700; Millipore) according to the manufacturer’s protocol. Briefly, Akata (EBV^+^) cells were either untreated or treated by IgG cross-linking for 24 h and then lysed with RIP lysis buffer from the kit. A part of the lysate (10%) was saved as input. The beads were washed with RIP wash buffer, followed by incubation with YTHDF2 antibody (Proteintech; 24744-1-AP) for 30 min at room temperature, and then washed twice with RIP wash buffer. The cell lysate was incubated with antibody-coated beads overnight at 4°C. The next day, the beads were collected and washed six times with RIP wash buffer. The enriched RNA-protein complex was treated with proteinase K, and the released RNA was purified using phenol-chloroform extraction and reverse transcribed for further qPCR analysis using primers listed in Table S1A.

### m^6^A RIP assay.

Total RNA was extracted from Akata (EBV^+^) cells (untreated or treated by IgG cross-linking for 24 h) with TRIzol (catalog no. 1596026; Thermo Scientific) and then treated with RQ1 DNase (catalog no. M6101; Promega) at 37°C for 30 min, followed by RNA re-extraction using TRIzol. Forty microliters of protein A/G magnetic beads (Thermo Scientific; 88802) was blocked in 1% bovine serum albumin (BSA) solution for 2 h, followed by incubation with 10 μg of anti-m^6^A antibody (Millipore; ABE572) at 4°C for 1 h with rotation. Three hundred micrograms of purified RNA was added to the antibody-bound beads in IP buffer containing 10 mM Tris-HCl at pH 7.4, 50 mM NaCl, and 0.1% NP-40 supplemented RNasin plus RNase inhibitor (catalog no. N2611; Promega) and incubated overnight at 4°C with rotation. The same RNA aliquot was used as input. The beads were washed three times with IP buffer, and RNA was eluted twice with IP buffer containing 6.67 mM m^6^A salt (catalog no. M2780; Sigma-Aldrich) (200 μl for each elution). The elutes were pooled and then purified with phenol-chloroform extraction. The immunoprecipitated RNA was reverse transcribed to cDNA for qPCR analysis. The primers were listed in Table S1A.

### Reporter cloning and luciferase assay.

To generate *CASP8*-exon 7 reporters, we used a psiCheck2 m^6^A-null vector, in which all putative m^6^A sites in the *Renilla* and firefly luciferase genes were mutated (52). The WT *CASP8*-exon 7, m^6^A Mut1 (5 conserved m^6^A sites were mutated to T), and Mut2 (all putative m^6^A sites were mutated to T) DNA fragments were synthesized using gBlocks (IDT) and were cloned into the psiCheck2 m^6^A null vector by Gibson assembly methods using primers and templates listed in Table S1A.

For luciferase assay, SNU-719 cells and SNU-719 YTHDF2-KD cells were seeded in 12-well plates prior to transfection. The cells were transfected with *CASP8*-exon 7 luciferase reporters using Lipofectamine 2000 reagent. Forty-eight hours posttransfection, cell extracts were harvested and measured using a dual-luciferase assay kit (Promega). Each condition was performed in triplicate.

### Bioinformatic analysis.

The multiz100way alignment prepared from 100 vertebrate genomes was downloaded from the UCSC Genome Browser (http://hgdownload.cse.ucsc.edu/goldenpath/hg38/multiz100way/). The nucleotide sequences around motifs of YTHDF1, YTHDF2, YTHDF3, and WTAP were extracted from multiz100way alignment by maf_parse of the Phast package (69). The corresponding amino acid sequences of each motif were inferred based on the nucleic acid sequence alignments. Sequences containing frameshifting indels were discarded for amino acid alignment to avoid ambiguity. Motif logos were then generated with WebLogo3 (70).

To analyze the conservation of *CASP8-*exon 7, we downloaded the phastCons 100-way conservation scores from 100 vertebrate genomes aligned to the human genome (http://hgdownload.cse.ucsc.edu/goldenpath/hg19/phastCons100way/). Given the m^6^A motif (DRACH), we obtained 15 putative m^6^A motif sequences within human *CASP8*-exon 7 while extracting corresponding sequences from 100 species. After removing sequences containing deletions within the m^6^A motif, we generated 15 motif logos by using the R package ggseqlogo (71).

### Quantification and statistical analysis.

Statistical analyses employed a two-tailed Student's *t* test using Microsoft Excel software for comparison of two groups. A *P* value less than 0.05 was considered statistically significant. Values are given as means and standard deviations (SD) for biological or technical replicate experiments, as stated in the figure legends.

### Data and material availability.

All the data needed to evaluate the conclusions in this paper are present in the paper and/or the supplemental materials. Additional data related to this paper may be requested from the authors.
